# Schwann cells regulate tumor cells and cancer-associated fibroblasts in the pancreatic ductal adenocarcinoma microenvironment

**DOI:** 10.1038/s41467-023-40314-w

**Published:** 2023-07-31

**Authors:** Meilin Xue, Youwei Zhu, Yongsheng Jiang, Lijie Han, Minmin Shi, Rui Su, Liwen Wang, Cheng Xiong, Chaofu Wang, Ting Wang, Shijie Deng, Dong Wu, Yizhi Cao, Lei Dong, Fan Bai, Shulin Zhao, Xiaxing Deng, Chenghong Peng, Hongwei Li, Jianjun Chen, Baiyong Shen, Lingxi Jiang, Hao Chen

**Affiliations:** 1 Department of General Surgery, Pancreatic Disease Center, Ruijin Hospital, Shanghai Jiao Tong University School of Medicine, Shanghai, China; 2 Research Institute of Pancreatic Diseases, Shanghai Jiao Tong University School of Medicine, Shanghai, China; 3 State Key Laboratory of Oncogenes and Related Genes, Institute of Translational Medicine, Shanghai Jiao Tong University, Shanghai, China; 4 Department of Systems Biology, Beckman Research Institute of City of Hope, Monrovia, CA 91016 USA; 5 Department of Pathology, Ruijin Hospital, Shanghai Jiao Tong University School of Medicine, Shanghai, China; 6 Biomedical Pioneering Innovation Center (BIOPIC), School of Life Sciences, Peking University, Beijing, China

**Keywords:** Cancer microenvironment, Pancreatic cancer

## Abstract

Neuropathy is a feature more frequently observed in pancreatic ductal adenocarcinoma (PDAC) than other tumors. Schwann cells, the most prevalent cell type in peripheral nerves, migrate toward tumor cells and associate with poor prognosis in PDAC. To unveil the effects of Schwann cells on the neuro-stroma niche, here we perform single-cell RNA-sequencing and microarray-based spatial transcriptome analysis of PDAC tissues. Results suggest that Schwann cells may drive tumor cells and cancer-associated fibroblasts (CAFs) to more malignant subtypes: basal-like and inflammatory CAFs (iCAFs), respectively. Moreover, in vitro and in vivo assays demonstrate that Schwann cells enhance the proliferation and migration of PDAC cells via Midkine signaling and promote the switch of CAFs to iCAFs via interleukin-1α. Culture of tumor cells and CAFs with Schwann cells conditioned medium accelerates PDAC progression. Thus, we reveal that Schwann cells induce malignant subtypes of tumor cells and CAFs in the PDAC milieu.

## Abstract (web summary)

The effects of Schwann cells on the neuro-stroma niche in pancreatic ductal adenocarcinoma (PDAC) remain to be explored. Here, single-cell RNA-sequencing and spatial transcriptome analysis of PDAC tissues reveal that Schwann cells induce malignant subtypes of tumour cells and cancer associated fibroblasts.

## Introduction

Although advances in cancer research over the past few decades have contributed to a steady increase in overall survival (OS) for many types of cancers, the prognosis of pancreatic ductal adenocarcinoma (PDAC) is still the worst in all cancers (5-years OS: 8–10%)^1^. The lack of a reliable test for early diagnosis and/or resistance to adjuvant therapy, are responsible for the dismal prognosis of PDAC^2^. PDAC is characterized by a dense fibrotic stroma which was composed of cancer-associated fibroblasts (CAFs), immunosuppressive cells, endothelial cells, and nerve cells. CAFs secrete tropic factors and extracellular matrix components, which lead to a low response rate to first-line chemotherapy^3^. Comparing with CAFs, limited studies have investigated nerve cells in the stroma of PDAC^4–6^.

Neuropathy is a representative character of PDAC, which contributes to pain, local recurrence, and worse prognosis in PDAC patients^7^. Ablation of nerves using neurotoxin capsaicin optimizes the prognosis of mice with PDAC^8^. Prominent perineural alterations were observed during PDAC progression, such as an increase in the size of intrapancreatic nerves (neural hypertrophy), neural density, and neural remodeling^9^. Growing evidence suggests a positive interaction between tumor cells and nerves. ADRB2-signaling pathway promotes the secretion of nerve growth factor (NGF) and brain-derived neurotrophic factor (BDNF) in PDAC cells, thereby increasing nerve density^10^. In contrast, as one of the major sources of acetylcholine (Ach), nerves activate cholinergic signaling in the gastric epithelium, inducing NGF expression and promoting carcinogenesis^11^. Elevated Ach from the vague nerve in PDAC reprograms the immune tumor microenvironment (TME) by epigenetic repression of CCL5 in neoplastic cells, and impairs the ability of PDAC cells to recruit CD8^+^ T cells^12^. Neurons not only secrete stimulatory factors to accelerate PDAC tumorigenesis but also provide metabolic support to PDAC cells by releasing serine^13^. Previous studies have predominantly focused on neurons, specifically the interactions between neurons, PDAC cells, and immune cells^8,13–16^. In addition to neurons, another key component of neuropathy is the Schwann cell, the predominant cell type in peripheral nerves. Remarkably, Schwann cells are detectable around pancreatic intraepithelial neoplasia (PanIN) lesions both in humans and mice^17^. Nonetheless, the Schwann cell’s contribution to the pancreas has not been explored. Schwann cells are crucial for neural repair and regeneration following nerve injury^18^. Similarly, after invasive malignant cells instigated nerve trauma, Schwann cells undergo a dynamic process of activation, which facilitates Schwann cells proliferation and migration to cancer cells^19^. In addition, Schwann cells migrate to malignant cells first before the latter invade the nerves^17^. Schwann cells exhibit a robust tropism for cancer cells and trigger nerve-cancer cell interactions^20^. Schwann cells actively participate in serious cancer processes, including cancer migration, invasion, immune exclusion, and transmission of cancer pain^21–24^. Through the NGF-Neurotrophic Receptor Tyrosine Kinase 1 (TrkA)-Nerve Growth Factor Receptor (NGFR) axis, Schwann cells exhibit robust chemotaxis towards neoplastic cells, in which participate in neural regeneration around cancer cells^17^. Inhibition of Schwann cell after cutaneous sensory nerve transection significantly decreased melanoma tumor development^25^. Emerging evidence suggests that Schwann cells promote cancer cell metastasis by secreting C-X-C Motif Chemokine Ligand 5 (CXCL5), Interleukin 6 (IL-6), and Transforming Growth Factor Beta 1 (TGF-β), or by direct cancer cell contact^26–29^. Active by c-Jun, Schwann cells could promote PDAC cancer cell migration and invasion^30^.

Moreover, Schwann cells could also play an important role in immunosuppressive TME by interacting with macrophages, mast cells, dendritic cells (DCs), myeloid-derived suppressor cells (MDSCs), and other immune cells^24,31^. Although the role of Schwann cells in cancer progression has been explored, the potential interactions between Schwann cells and other main components of the TME, especially CAFs in PDAC, is limited. The mechanism by which Schwann cells regulate PDAC TME is yet to be elucidated.

In this work, immunohistochemistry (IHC) staining of PDAC tissue microarray (TMA) shows the location of Schwann cells in tumors. Further, integrated single-cell RNA sequencing (scRNA-seq) and microarray-based spatial transcriptomics (ST) reveal the heterogeneity of tumor cells and CAFs adjacent to Schwann cells. Furthermore, in vitro and in vivo functional assays and pharmacological inhibition validate the malignant function and the underlying mechanism of Schwann cells.

## Results

### Prevalence of Schwann cells accumulation in the TME and its clinical significance

**Fig. 1:**
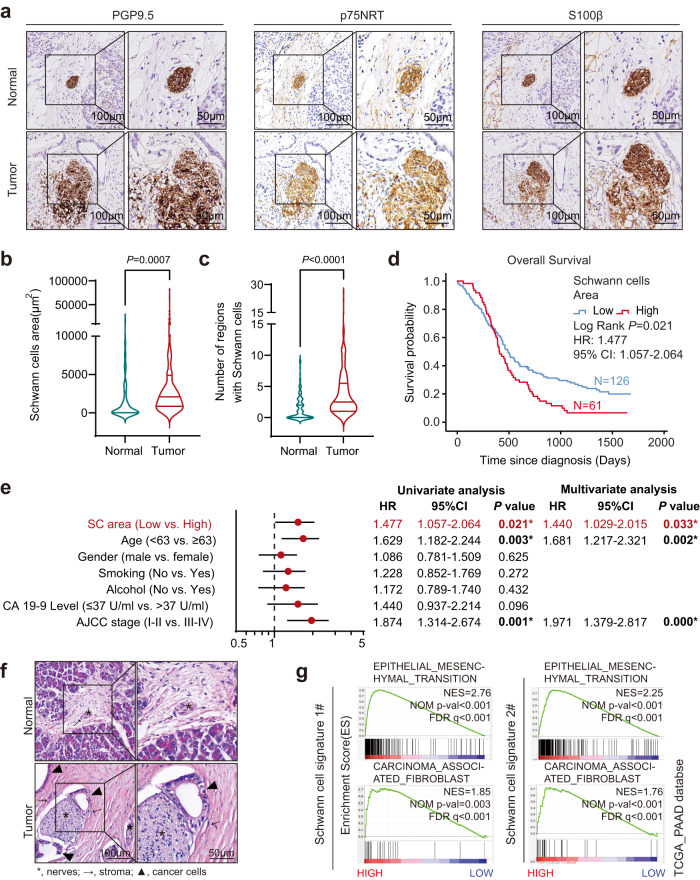
Prevalence of Schwann cell accumulation in the TME and its clinical significance. **a** Representatives of IHC staining images with anti-PGP9.5, p75NRT, and S100β. Scale bar, left, 100 µm; right, 50 µm. Images are representative of 187 PDAC samples with similar results. **b**, **c** Violin chart of Schwann cells area (**b**) or the number of regions used for calculating Schwann cells area (**c**). For each violin, the minimum, first quartile, median, third quartile, and maximum were displayed. *n* = 187. **d** OS analysis of PDAC patients with low Schwann cells area (<3190 μm^2^; *n* = 126) or high area (≥3190 μm^2^; *n* = 61) in PDAC patients (*n* = 187). The cutoff of Schwann cells area was determined by X-Tile^76^. HR and 95% CI were determined using the regression coefficient of the Cox model. **e** Univariate and multivariate analysis of the OS of PDAC patients (*n* = 187). The dot of the Forest plot represents the hazard ratio of the Cox proportional hazards model, the error bars are two-sided 95% confidence intervals. *P < 0.05. Independent variables with *P* < 0.05 in the univariate analysis were included in the multivariate analysis. **f** H&E staining showing nerves, stroma, and cancer cells in PDAC tumor and normal tissue. asterisk, nerves; arrow, stroma; triangle, cancer cells. Images are representative of three PDAC samples with similar results. Scale bar, left, 100 µm; right, 50 µm. **g** GSEA plot showing that EMT and carcinoma-associated fibroblasts were enriched in PDAC samples with higher expression of Schwann cell signatures. Pathway enrichment analysis was performed using data from the TCGA-PAAD cohort. NES normalized enrichment score, corrected for multiple comparisons using FDR method, *P* value were showed in plots. Statistical analysis: unpaired two-sided *t*-test (**b**, **c**); log-rank test (**d**). Source data are provided as a Source Data file.

To unveil the distribution of Schwann cells in PDAC, the pan-neural marker Ubiquitin C-Terminal Hydrolase L1 (PGP9.5) and three traditional Schwann cell markers, nerve growth factor receptor (p75NRT), S100 Calcium Binding Protein B (S100β) and glial fibrillary acidic protein (GFAP)^17,29^, were used for IHC staining and immunofluorescence (IF) in a cohort of PDAC patients (*n* = 187). Markers of Schwann cells (p75NRT, S100β, and GFAP) were co-localized with PGP9.5, suggesting that the intrapancreatic nerves were accompanied by Schwann cells (Fig. 1a and Supplementary Fig. S1a, b). Histological sections showed that Schwann cells were enriched in PDAC compared to non-tumor tissues (Fig. 1b, c). As expected, the intrapancreatic area of Schwann cells was increased; this was associated with worse prognosis for PDAC (*P* = 0.021, Fig. 1d); and could be used as an independent prognostic factor for OS (HR = 1.440, 95% CI: 1.029–2.015; *P* = 0.033) (Fig. 1e). Using hematoxylin and eosin (H&E) staining, we observed that Schwann cells were surrounded by stromal and tumor cells (Fig.1f and Supplementary Fig. S1c). Bioinformatic analysis of bulk RNA-seq data from The Cancer Genome Atlas (TCGA)-PAAD cohort revealed that significant activation of epithelial-mesenchymal transition (EMT) and carcinoma-associated fibroblasts were enriched in tumor tissues with high expression of each Schwann cell signature^32–34^ (Fig.1g and Supplementary Fig. S1d), suggesting that Schwann cells might communicate with tumor cells and fibroblasts and promote malignant progression in PDAC.

### Single-cell analysis reveals the heterogeneity of tumor cells and CAFs in PDAC with Schwann cells accumulation

**Fig. 2:**
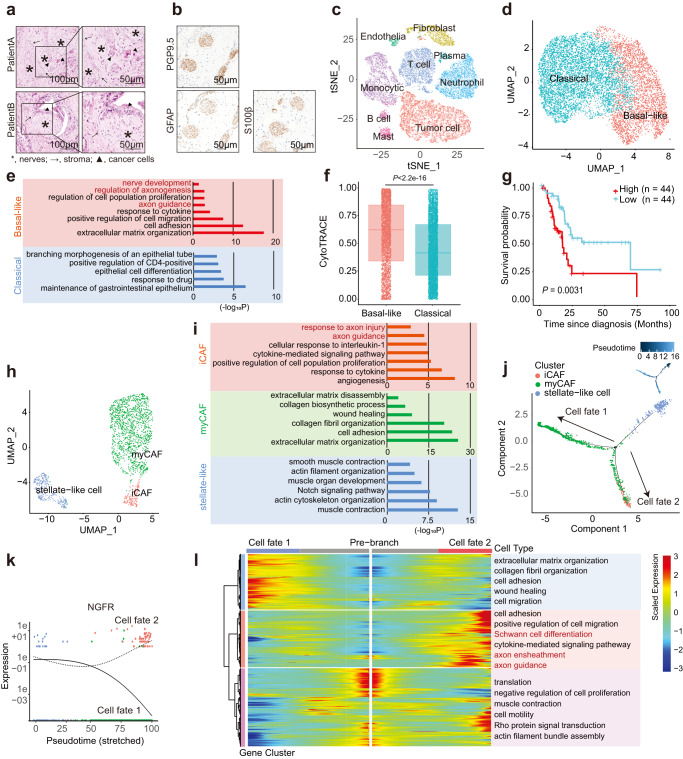
Single-cell analysis reveals the heterogeneity of tumor cells and CAFs in PDAC with Schwann cell accumulation. **a** Representative H&E images of PDAC tissues with neural hypertrophy. asterisk, nerves; arrow, stroma; triangle, cancer cells. Scale bar, left, 100 µm; right, 50 µm. **b** Representatives of IHC staining images of patient A with anti-PGP9.5, GFAP, and S100β. Scale bar, 50 µm. **a**, **b** Images are representative of four PDAC samples with similar results. **c** *t*-SNE plot depicting the major cell types identified by single-cell RNA sequencing of four PDAC tissues. **d** UMAP plot showing two main subclusters, basal-like and classical tumor cells from PDAC tissues. **e** GO analysis showing the upregulated pathways in basal-like and classical tumor cells. **f** The differentiation states of cancer cells defined by CytoTRACE. For each boxplot, the first quartile, median, and third quartile ± s.d were displayed. *n* = 4 PDAC patients. **g** OS analysis of patients with low (*n* = 44) or with high (*n* = 44) basal-like signature in the TCGA-PAAD cohort (*n* = 88). **h** UMAP plot showing three main subclusters of fibroblasts, including iCAFs, myCAFs, and stellate-like cells. **i** GO analysis showing the upregulated pathways in iCAFs, myCAFs, and stellate-like cells. **j** Trajectory of fibroblasts along pseudotime in a two-dimensional space. **k** Expression of *NGFR* in the two cell fates of fibroblasts along pseudotime. The expression of *NGFR* was determined by branch expression analysis. **l** Heatmap showing the dynamic changes of gene expression along pseudotime. The differentially expressed genes were clustered hierarchically into three groups and the representative enriched pathways of each group were shown. Statistical analysis: one-sided Fisher’s exact test (**e**, **i**); two-sided Wilcoxon rank-sum test (**f**); log-rank test (**g**).

In the nervous system, Schwann cells play a substantial role in maintaining homeostasis and possess the capacity for inflammation and regeneration^35^. To explore whether Schwann cells shape the heterogeneity of tumor cells and CAFs in the neuro-stroma milieu, scRNA-seq was applied to four PDAC tissues with Schwann cells accumulation, which was confirmed by tumor histopathology and IHC (Fig. 2a, b and Supplementary Fig. S2a, b). A total of 25,150 high-quality cell profiles were obtained for downstream analysis. Nine major cell types were characterized: tumor cells, fibroblasts, neutrophils, monocytes, T cells, B cells, mast cells, plasma cells, and endothelial cells (Fig.2c; Supplementary Fig. S2c; and Supplementary Data 1). Tumor cells and fibroblasts were selected for further experiments.

Tumor cells formed two distinct subclusters with unique gene signatures upon uniform manifold approximation and projection (UMAP) analysis and were annotated as basal-like and classical-like signatures previously described in PDAC^36–38^ (Fig. 2d, Supplementary Fig. S2c, d, and Supplementary Data 1). The classical subtype showed higher expression of the epithelial pathway, and the basal-like subtype was enriched in the basal-related pathway, as previously described^37,38^ (Fig. 2e and Supplementary Fig. S2e). Interestingly, the basal-like subtype was also enriched in nerves corresponding signaling pathways such as axon guidance, regulation of axonogenesis, and nerve development (Fig. 2e). Cellular (Cyto) trajectory reconstruction analysis using gene counts and expression (CytoTRACE)^39^ revealed that tumor cells with a high basal-like signature score displayed poor differentiation compared to those with a classical signature (Fig. 2f; Supplementary Fig. S2f, g; and Supplementary Data 1). Consistent with previous studies, the basal-like signature was an unfavorable prognostic factor in TCGA-PAAD cohort^38,40,41^ (Fig. 2g).

Although the existence of CAF heterogeneity has been established, the phenotypic characteristics of CAFs in the neuro-stroma environment remain unexplored. Unsupervised clustering analysis showed that fibroblasts were categorized as stellate-like cells and CAFs. CAFs were further identified as a myofibroblastic subset (myCAF) and inflammatory subset (iCAF), according to previous studies (Fig. 2h; Supplementary Fig. S2c, h, i; and Supplementary Data 1)^6,32,33,42^. The pathways of ECM organization and cell adhesion were enriched in the myCAF cluster, whereas the pathways related to muscle contraction and actin cytoskeleton were enriched in stellate-like cells (Fig. 2i and Supplementary Fig. S2j). Pancreatic stellate cells (PSCs) functional genes^32,33^ were enriched in stellate-like cells, including *FABP4*, associated with lipid transport and retinoid storage, and *ADIRF*, associated with adipogenesis (Supplementary Fig. S2i).

Notably, the axon guidance pathway and *NGFR*, which could bind to NGF and other neurotrophins, were also activated in the iCAF cluster (Fig. 2i and Supplementary Fig. S2j, k). To further investigate the potential transitional process of CAFs in TME, monocle2 was applied to construct the pseudotime map of the fibroblast developmental state trajectory. Consistent with most studies reported that PSCs were the major source of CAFs in PDAC^43^, stellate-like cells were the start of trajectories. iCAFs featured terminal differentiation and were considered the end of one trajectory (Fig. 2j and Supplementary Data 1). Moreover, nerve-corresponding genes, including *NGFR*, were expressed dynamically with pseudotime in CAFs (Fig. 2k and Supplementary Data 1). Branched expression analysis modeling (BEAM) was performed to investigate the two branches of CAFs^44^. The dynamic relationship between stellate cells, iCAFs, and myCAFs further confirmed that matrix organization-related genes were activated in myCAFs destined for cell fate 1, while cell migration, nerves, and Schwann cell corresponding pathways were enriched in iCAFs and the other myCAFs for cell fate 2 (Fig. 2l). Together, the PDAC milieu with neural hypertrophy and Schwann cells accumulation consisted of malignant clusters of CAFs and tumor cells, which may participate in nerve development and Schwann cell recruitment during disease progression.

Similar to previous studies, we only detected a small Schwann-like cells cluster (four cells, 0.0159%), with *CDH19*, *PLP1*, and *SOX10* high expression^32,33^ (Supplementary Fig. S2l). However, due to the scarcity of cell numbers, no further investigation was performed on these putative Schwann cells. Alternatively, a spatial transcriptome was performed to explore the location and heterogeneity of cell types in the neuro-stroma niche.

### Microarray-based spatial transcriptomics reveals that Schwann cells are surrounded by iCAFs and basal-like tumor cells

**Fig. 3:**
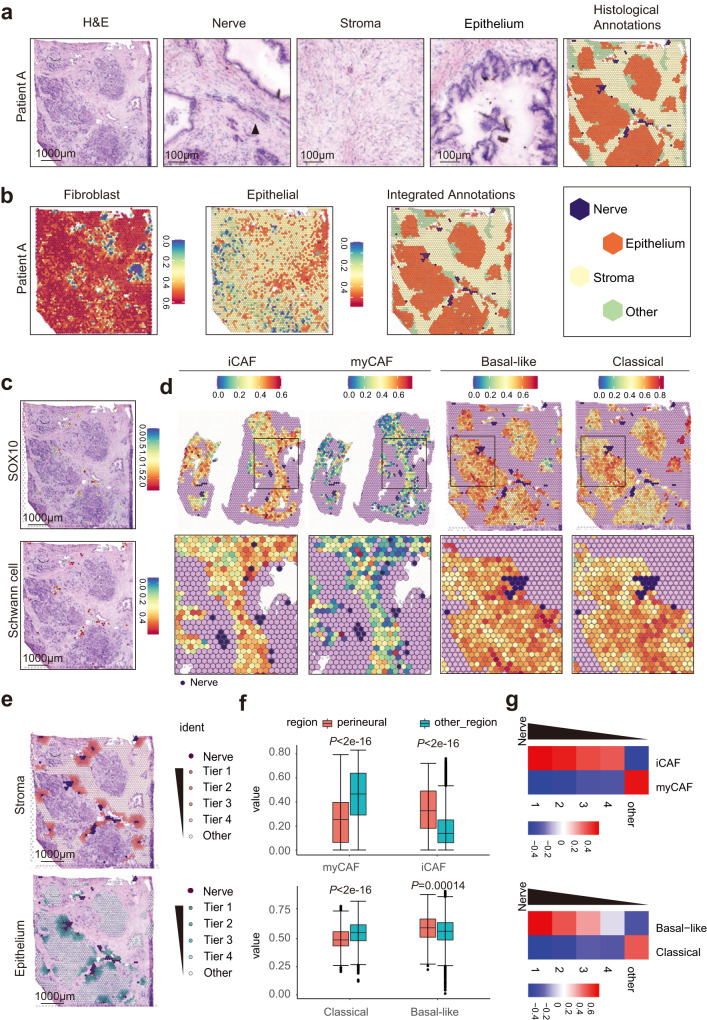
Microarray-based spatial transcriptomics reveals that Schwann cells are surrounded by iCAFs and basal-like tumor cells. **a** Histological annotations of tumor cryosection from patient A. Images are representative of four PDAC samples with similar results. Scale bar, left, 1000 µm; right, 100 µm. **b** Stroma and epithelium regions were defined by integrating the results of ssGSEA and histologic annotation. **c** Schwann cell markers SOX10 was co-localized with nerve region in Patients A (upper panel). The score of Schwann cell signature determined by ssGSEA is high in nerve regions (lower panel). Scale bar, 1000 µm. **d** The scores of iCAF, myCAF, basal-like and classical signatures determined by ssGSEA in the neuro-stroma niche. **e** Perineural tier 1‒4 and other regions were defined according to the distance to a nerve in the neuro-stroma niche. Scale bar, 1000 µm. **f** The ssGSEA scores of iCAF, myCAF, basal-like and classical signature in the perineural region (tier 1) compared with the other regions (tier 2‒4 and other regions). For each boxplot, the first quartile, median, and third quartile ± s.d were displayed. Patients number = 4, nerves number = 40. **g** The QuSAGE scores of iCAF, myCAF, basal-like, and classical signature in tier 1‒4 and other regions in the neuro-stroma niche. Statistical analysis: two-sided Wilcoxon rank-sum test (**f**).

A series of cryosections of four fresh PDAC tissues were used for spatial transcriptome (ST) analysis. H&E staining was performed to validate the accurate location of nerves, stroma, and epithelium. The regions of the nerves, stroma, and epithelium were annotated in each slide according to the histological features (Fig. 3a and Supplementary Fig. S3a). Single-sample gene set enrichment analysis (ssGSEA) was performed to quantify the spatial distribution of fibroblasts and tumor cells using the corresponding markers in scRNA-seq (Fig. 3b and Supplementary Fig. S3b). By integrated analysis of the two annotations, the regions of the stroma and epithelium, were determined for the following analysis (Fig. 3b and Supplementary Fig. S3b). Unsupervised clustering analysis identified cluster 19 with high expression of Schwann cell-related pathways and markers (SOX10 and GAP-43) (Fig. 3c; Supplementary Fig. S3c–f; and Supplementary Data 2). As expected, most nerve regions overlapped with the regions of Cluster 19 and high ssGSEA score of Schwann cells signature was also enriched in nerve regions (Fig. 3c).

Further, ssGSEA of iCAFs, myCAFs, basal-like tumor cells, and classical tumor cells was performed. Strikingly, the nerve regions were surrounded by regions with high scores of iCAFs and basal-like signatures, whereas regions far away from the nerve were enriched with high scores of myCAFs and classical signatures (Fig. 3d and Supplementary Data 2). Furthermore, the perineural region was divided into four tiers according to the distance to the nerve (Fig. 3e and Supplementary Fig. S3g), and quantitative set analysis of gene expression (QuSAGE) was performed in each tier^45^. We found that iCAFs and basal-like signatures progressively increased from distant to proximal regions of the nerve, whereas myCAFs and classical signatures decreased (Fig. 3f, g). The heatmap showed that a similar change of marker genes for iCAF, myCAF, basal-like tumor cell, and classical tumor cell signatures (Fig. 3g and Supplementary Fig. S3h). Similar results were obtained using the reported markers of CAFs and tumor cells in previous studies^6,46^ (Supplementary Fig. S3i). Collectively, the subtypes of CAFs and tumor cells adjacent to Schwann cells were characterized.

### Schwann cells promote malignant progression in PDAC via midkine (MDK)

**Fig. 4:**
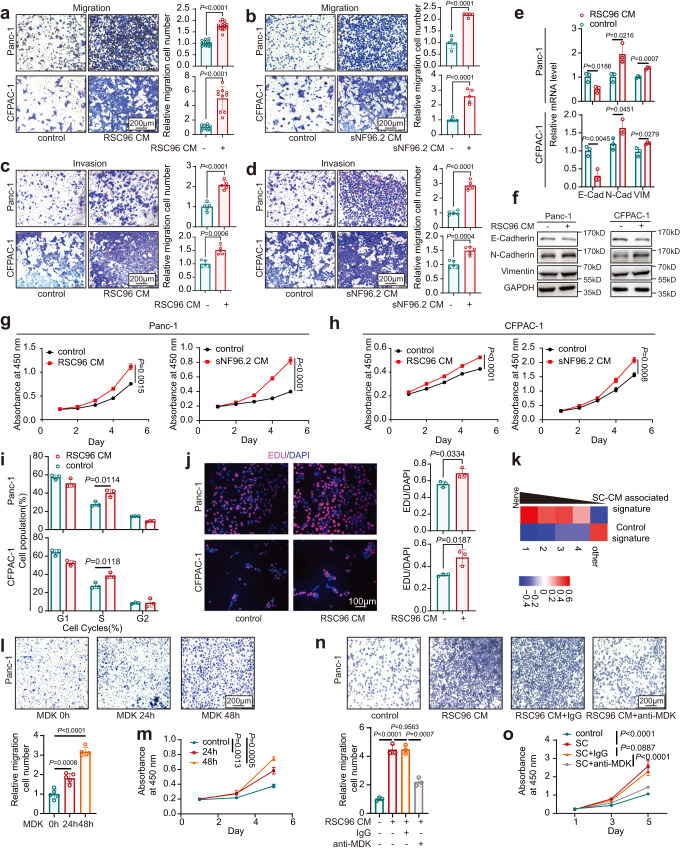
Schwann cells promote malignant progression in PDAC via MDK. **a**–**d** Effects of RSC96 CM (**a**, **c**) and sNF96.2 CM (**b**, **d**) on migration (**a**, **b**) and invasion (**c**, **d**) of Panc-1 and CFPAC-1 cells were assessed by Transwell and Matrigel invasion assays. *n* = 10, 15, 16 (**a**) or 5 (**b**–**d**) representative pictures over three independent experiments. Scale bar, 200 µm. **e**, **f** The relative expression levels of EMT markers in Panc-1 and CFPAC-1 cells cultured with RSC96 CM were detected by qPCR (**e**) and western blotting (**f**). Data were representative of *n* = 3 independent experiments. **g**–**j** Effects of RSC96 CM and sNF96.2 CM on the cell proliferation of Panc-1 and CFPAC-1 cells were assessed by CCK-8 (**g**, **h**), flow cytometry (**i**), and EdU assays (**j**). Scale bar, 100 µm. **k** QuSAGE scores of control and SC-CM associated signature in perineural tier 1‒4 and other regions in the neuro-stroma niche. The signatures were based on the bulk RNA-seq of CFPAC-1. **l**, **m** Effects of recombinant human MDK on migration (**l**) and proliferation (**m**) of Panc-1 cells were assessed by Transwell (**l**) and CCK-8 assay (**m**). Scale bar, 200 µm. **n**, **o**. Effects of MDK neutralization antibodies on migration (**n**) and proliferation (**o**) of Panc-1 cells cultured with RSC96 CM were assessed by Transwell (**n**) and CCK-8 (**o**) assays. Scale bar, 200 µm. **e**, **g**–**j**, **m**, **n** Data were the mean ± s.d. of *n* = 3 independent experiments. **l**, **o** Data were the mean ± s.d. of *n* = 5 independent experiments. Statistical analysis: unpaired two-sided *t*-test (**a**–**e**, **i**, **j**, **l**, **n**); two-way ANOVA (**g**, **h**, **m**, **o**). Source data are provided as a Source Data file.

ST results showed that the basal-like signature was upregulated in the surrounding Schwann cells (Fig. 3f, g and Supplementary Fig. S3h, i). It was widely recognized that basal-like cells are the highly aggressive subtype in PDAC. To explore whether Schwann cells facilitate PDAC cells gain malignant phenotypes, Schwann cell-conditional medium (SC-CM) of two cell lines (RSC96 and sNF96.2) was used to culture PDAC cells. Consistent with a previous study^28^, the migration and invasion abilities of PDAC cells were enhanced by SC-CM incubation (Fig. 4a–d). Elevated expression of EMT markers was also observed after SC-CM treatment (Fig. 4e, f). Similar results were observed for cell proliferation and the cell cycle (Fig. 4g–j and Supplementary Fig. S4a). As expected, GSEA indicated that EMT and cell cycle pathways were enriched in groups treated with SC-CM compared to controls (Supplementary Fig. S4b). Consistent with our scRNA-seq data, upregulation of basal-like-related signatures and downregulation of classical-related signatures were also shown in the CM treatment group via GSEA (Supplementary Fig. S4c). The gene set upregulated by SC-CM was defined as the SC-CM-associated signature, which exhibited a similar trend to basal-like signatures according to the distance to the nerve in ST data (Figs. 3g, 4k and Supplementary Table S1).

Mass spectrometry (MS) was performed to identify the key components of SC-CM in promoting malignant progression. Of note, among the ligands identified in SC-CM of all used cell lines, MDK aroused our interest as it is a neurite growth-promoting factor proposed to mediate metastasis in several cancer types (Supplementary Fig. S4d and Supplementary Data 3)^47–49^. Recombinant MDK was used to treat PDAC cells to assess the effects of MDK on tumor progression. Similar to SC-CM treatment, MDK treatment also significantly enhanced the cell proliferation and migration capacities of PDAC cells (Fig.4l, m). Moreover, the pro-tumor functional role of Schwann cells could be greatly compromised by neutralizing antibodies against MDK (Fig. 4n, o). Thus, these results demonstrated that Schwann cells promote the malignant progression of PDAC via MDK.

### Schwann cells induce a phenotypic switch in CAFs via interleukin-1 alpha (IL-1α)

**Fig. 5:**
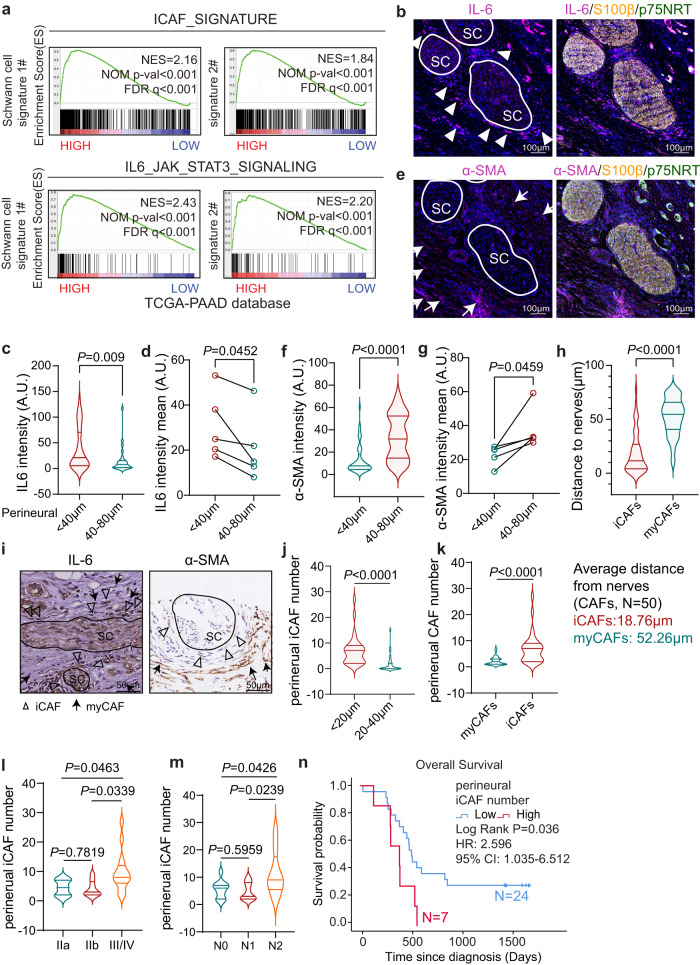
Perineural iCAFs widely exist in PDAC. **a** GSEA plot showing that the iCAF signature and IL6_JAK_STAT3 pathway were enriched in PDAC samples with high expression levels of Schwann cell signature. **b** Representative images of IF with anti-IL-6 (a marker for iCAFs) and S100 β/p75NRT (a marker for Schwann cells) in the neuro-stroma niche. Scale bar, 100 µm. **c**, **d** Quantification of IL-6 intensity in the regions of 0 to 40 μm or 40 to 80 μm away from Schwann cells in representative panel **b** (**c**) or mean intensity of five independent PDAC patients (**d**). **e** Representative images of IF with anti-α-SMA (a marker for myCAFs) and S100 β/p75NRT in the neuro-stroma niche. Scale bar, 100 µm. **b**, **e** Images are representative of five PDAC samples with similar results. **f**, **g** Quantification of α-SMA intensity in representative panel **e** (**f**) or mean intensity of five independent PDAC patients (**g**). **h** Quantification of the average distance of iCAFs or myCAFs to Schwann cells. *n* = 50 cells. **i** Representative images of IHC staining with anti-IL-6 and α-SMA in human PDAC. Scale bar, 50 µm. Images are representative of 31 PDAC samples with similar results. **j** Quantification of the iCAFs (IL-6^high^/α-SMA^Low^) number 0 to 20 or 20 to 40 μm away from Schwann cells in 31 PDAC patients. **k** Quantification of the iCAFs or myCAFs (IL-6^Low^/α-SMA^High^) number in the region of 0 to 20 μm away from Schwann cells in 31 PDAC patients. **l** Violin chart of iCAFs number of PDAC with AJCC stage (IIa, *n* = 8; IIb, *n* = 8; III/IV *n* = 15) in 31 PDAC patients. **m** Violin chart of iCAFs number of PDAC with N stage (N0, *n* = 11; N1, *n* = 11; N2, *n* = 9) in 31 PDAC patients. **c**, **f**, **h**, **j**, **k**, **l**, **m** For each violin, the minimum, first quartile, median, third quartile, and maximum were displayed. **n** OS analysis of PDAC patients with low perineural iCAF number (<8; *n* = 24) or high (>9; *n* = 7) in PDAC patients (*n* = 31). Cutoff of the iCAF number was determined by X-tile. HR and 95% CI were determined using the regression coefficient of the Cox model. Statistical analysis: unpaired two-sided *t*-test (**c**, **f**, **h**, **j**–**m**); paired two-sided *t*-test (**d**, **g**); log-rank test (**n**). Source data are provided as a Source Data file.

Cancer progression is recognized as a result of evolving crosstalk between cancer cells and the surrounding CAFs, especially in PDAC, which is characterized by prominent cancer-associated desmoplasia^2,50^. In addition to basal-like tumor cells, iCAFs were also observed adjacent to Schwann cells in the ST data (Fig. 3f, g and Supplementary Fig. S3h, i). Moreover, bioinformatics analysis of bulk RNA-seq data from the TCGA cohort revealed that Schwann cell signature expression exhibited significant enrichment of the iCAF-related signature (Fig. 5a and Supplementary Fig. S5a). We first performed multiple IF staining to verify the spatial distribution of iCAFs and myCAFs in PDAC tumor tissues. The results revealed that perineural IL-6^high^ cells (a marker of iCAFs^43^) were adjacent to Schwann cells, whereas α-SMA ^high^ cells (a marker of myCAFs^43^) were located farther away from the neuro-stroma niche (Fig. 5b–g and Supplementary Fig. S5b). The average distance of iCAFs/myCAFs from the nerves turns to be 18.76 and 52.26 μm, respectively (Fig. 5h). We further demonstrated the spatial distribution of iCAFs/myCAFs/nerves in clinical PDAC samples (*n* = 31) using IHC staining. We also detected the perineural iCAFs and EMT cancer cells enrichment, consistently (Fig. 5i–k and Supplementary Fig. S5c–h). Higher perineural iCAF numbers correlated with a more aggressive tumor stage and worse patient outcome (Fig. 5l–n). Thus, we hypothesized that Schwann cells might shape the surrounding CAFs and induce the switch of phenotypes, which further lead to PDAC progression.

**Fig. 6:**
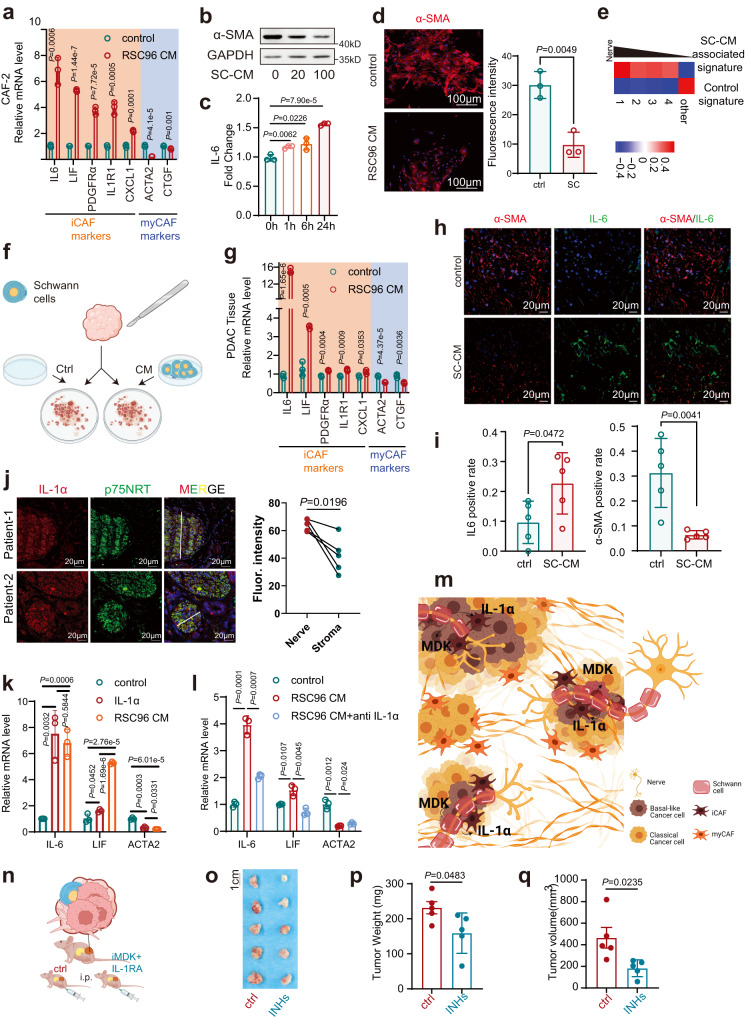
Schwann cells induce a phenotypic switch in CAFs via IL-1α. **a** The relative mRNA levels of iCAF and myCAF markers in CAF-2 cultured with RSC96 CM and control. **b** The protein level of α-SMA in CAF-2 cultured with 0, 20, or 100 μg/ml RSC96 CM. **c** The fold change of IL-6 in CAF-2 cultured with RSC96 CM for 0, 1, 6, or 24 h was evaluated by ELISA. **d** Representative images and quantification of IF with anti-α-SMA in CAF-2 cultured with RSC96 CM and control. Scale bar, 100 µm. Images are representative of three biologically independent experiments with CAF-2 cells with similar results. **e** QuSAGE scores of control and SC-CM associated signature in perineural tier 1–4 and other regions in the neuro-stroma niche. The signatures were defined by the bulk RNA-seq of CAFs with SC-CM treatment. **f** Schematic diagram of ex vivo assay. **g** The relative mRNA levels of iCAF and myCAF markers in PDAC tissue cultured with RSC96 CM and control. **h**, **i** Representative images (**h**) and quantification (**i**) of IF with anti-α-SMA and IL-6 in PDAC tissue cultured with RSC96 CM and control. Scale bar, 20 µm. **j** Representative images and quantification of IL-1α in PDAC tissues. Scale bar, 20 µm. **h**, **j** Images are representative of five PDAC samples with similar results. **k** The relative mRNA levels of iCAF and myCAF markers in CAF-2 treated with IL-1α or RSC96 CM. **l** The relative mRNA levels of iCAF and myCAF markers in CAF-2 cultured by RSC96 CM pretreated with neutralization antibodies against IL-1α or IgG control. **m** Schematic diagram of how Schwann cells shape tumor cells and CAFs in the neuro-stroma niche. **n** Schematic diagram of the in vivo orthotopic injection. A cell mixture of CAFs, CFPAC-1, and sNF96.2 cells was injected into mice pancreas, then the mice subjected to MDK inhibitor (iMDK) and interleukin-1 receptor antagonist (IL-1RA) treatment (INHs, inhibitors) or not (*n* = 5). **o**–**q** Photograph (**o**) and quantification (**p**, **q**) of orthotopic tumors of mice. **a**, **c**, **d**, **g**, **k**. **l** Data were the mean ± s.d. of *n* = 3 independent experiments. **i**, **p**, **q** Data were the mean ± s.d. of *n* = 5 independent experiments. **f**, **m**, **n** Created with BioRender.com. Statistical analysis: unpaired two-sided *t*-test (**a**, **c**, **d**, **g**, **i**, **k**, **l**, **p**, **q**); paired two-sided *t*-test (**j**). Source data are provided as a Source Data file.

To further characterize the role of Schwann cells in CAFs, SC-CM was applied to fresh PDAC tissues derived CAFs (CAF-1 and CAF-2), which were then cultured as primary cells and validated using western blotting and IF (Supplementary Fig.S6a, b). SC-CM incubation promoted the proliferation of CAFs, as the percentage of cells in the S phase was significantly increased, and the apoptotic index was markedly decreased in the treatment group compared to the control group (Supplementary Fig. S6c–f). Gene ontology (GO) and Kyoto Encyclopedia of Genes and Genomes (KEGG) analyses of RNA-seq for CAFs showed that the pathways of cytokines, cell cycle, and nerve system development were enriched in CAFs after SC-CM incubation (Supplementary Fig. S6g). Next, whether Schwann cells induced the switch of CAF to iCAF phenotype was studied; SC-CM incubation resulted in the upregulation of iCAF markers (IL-6, LIF, PDGFRα, IL1R1, and CXCL1) and the downregulation of myCAF markers (α-SMA and CTGF) in CAFs (Fig.6a–d and Supplementary Fig. S7a–d). Similar results were observed with the co-culture of CAFs and Schwann cells (Supplementary Fig. S7e). Consistent with our scRNA-seq results, SC-CM treatment could also switch PSCs, the major source of CAFs in PDAC, to the iCAF phenotype (Supplementary Fig. S7f–h). Interestingly, SC-CM incubation upregulated the pathways of cytokine-cytokine receptor interaction and neuroactive ligand–receptor interaction in CAFs, which was an echo of the features of iCAFs unveiled in scRNA-seq (Supplementary Fig. S7i). As expected, the SC-CM-associated CAF signature displayed a similar trend in the iCAF signature according to the distance to the nerve (Figs. 3g, 6e and Supplementary Table S1).

To further recapitulate the reversible features of CAFs in primary PDAC tissues, a three-dimensional ex vivo culture model of tumor tissues was established (Fig. 6f and Supplementary Fig. S7j). Fresh PDAC tissues were dissected into approximately 1 mm^3^ pieces and then submerged in a culture medium with or without SC-CM^51^. Increased levels of inflammatory cytokines and loss of myCAF features were observed in ex vivo tumor tissues (Fig. 6g–i and Supplementary Fig. S7k, l). Moreover, GESA of RNA-seq results showed that SC-CM incubation induced the upregulation of iCAF-related signatures while decreasing the signatures of myCAFs (Supplementary Fig. S7m). Thus, Schwann cells could trigger CAFs to switch to the iCAFs phenotype. IL-1α is the major ligand responsible for iCAF formation in PDAC^52^. It was intriguing to find that Schwann cells were an important source of IL-1α (Fig. 6j and Supplementary Fig. S8a–d). Similar to SC-CM, recombinant IL-1α upregulated the markers of iCAFs, whereas neutralizing antibodies against IL-1α abolished the effects of Schwann cells on iCAF phenotypes (Fig. 6k, l and Supplementary Fig. S8e–g). Together, our data revealed a malignant niche of PDAC, in which Schwann cells shape the heterogeneity of CAFs and cancer cells. Schwann cells enhance basal-like features in PDAC cells via MDK. Besides, Schwann cells induce the switch of CAFs to iCAFs by secreting IL-1α. (Fig. 6m).

To further validate the therapeutic potential of nerve/stroma/tumor niches, an orthotopic Schwann cells-containing tumor was induced by co-injection of PDAC cells, CAFs, and Schwann cells to mice pancreas (Fig. 6n). In consistent, MDK inhibitor (iMDK) and Interleukin-1 receptor antagonist (IL-1RA) substantially suppressed PDAC progression (Fig. 6n–q and Supplementary Fig. S8h).

### Schwann cells hijack iCAFs to accelerate cancer progression

Co-culture of tumor organoids and iCAFs leads to a marked upregulation of EMT markers and iCAFs are associated with a poor prognosis^43,50,52,53^. To investigate whether iCAFs induced by Schwann cells contribute to tumor progression, in vitro and in vivo functional assays of PDAC cells were performed.

**Fig. 7:**
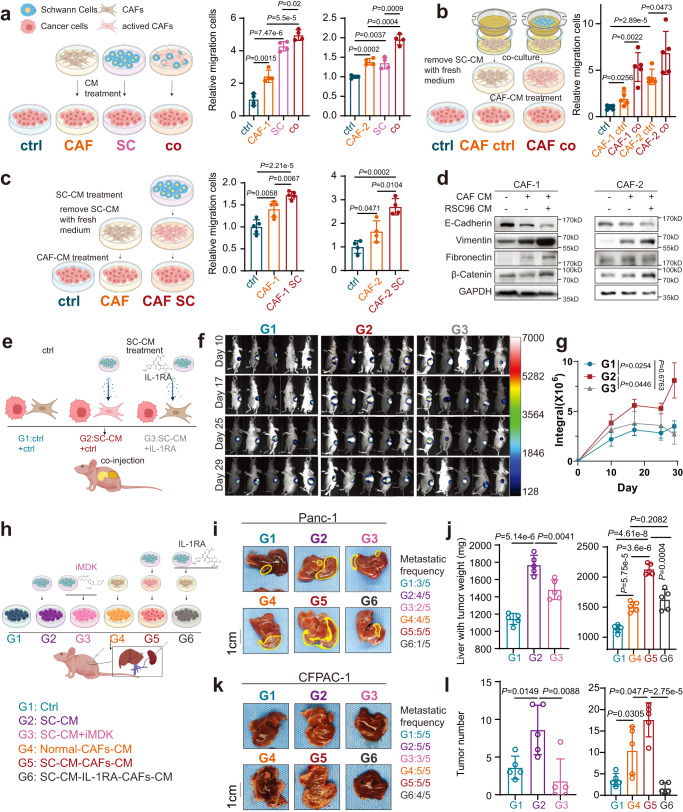
Schwann cells hijack iCAFs to accelerate cancer progression. **a**‒**c** The migration capabilities of Panc-1 cells were enhanced by the CM of activated CAFs. CAFs were directly co-cultured (**a**) or Transwell co-cultured with (**b**) Schwann cells, or cultured with Schwann cells CM (**c**), respectively. Direct co-culture (**a**): CM of CAFs, RSC96, and cell mixture of CAFs and RSC96 were harvested. Transwell co-culture (**b**): CAFs and RSC96 cells were co-cultured via Transwell chambers for 48 h, and CAFs was washed by PBS three times, then CAFs were cultured with fresh medium for another 48 h, at last, CAFs CM was harvested. Schwann cell CM (**c**): CAFs was cultured with Schwann cell CM for 48 h and the removal of SC-CM was followed by three rinses with PBS, and then CAFs were cultured with fresh medium for another 48 h, at last, CAFs CM was harvested. After 48 h culture with CAFs CM, Panc-1 cells were harvested for migration assay. **d** The protein levels of E-cadherin, Vimentin, Fibronectin, and β-catenin in Panc-1 cells treated with CM of CAFs, which were cultured with or without (w/ or w/o) RSC96 CM. Data were representative of *n* = 3 independent experiments. **e** Schematic diagram of the in vivo orthotopic injection. A cell mixture of CAFs (w/ or w/o RSC96 CM/IL-1RA treatment) and tumor cells was injected into mice pancreas (*n* = 5). **f**, **g** Bioluminescence photograph (**f**) and quantification (**g**) of orthotopic tumors of mice injected with a cell mixture of Panc-1 cells and CAFs. **h** Schematic diagram of in vivo liver metastatic model. Panc-1 (**i**, **j**) or CFPAC-1(**k**, **l**) were pretreated with ctrl medium (G1), SC-CM(G2), SC-CM+iMDK(G3), ctrl-CAF-CM(G4), SC-induced CAF-CM(G5), or SC + IL-1RA-induced CAF-CM (G6), and then injected via spleen (*n* = 5). **i**–**l** Representative photograph (**i**, **k**) and quantification (**j**, **l**) of Panc-1 (**i**, **j**) or CFPAC-1 (**k**, **l**) liver metastatic tumors. **a**, **c** (right), Data were the mean ± s.d. of *n* = 4 independent experiments. **b**, **c** (left), **g**, **j**, **l** Data were the mean ± s.d. of *n* = 5 independent experiments. **a**–**c**, **e**, **h** Created with BioRender.com. Statistical analysis: unpaired two-sided *t*-test (**a**–**c**, **j**, **l**); two-way ANOVA (**g**). Source data are provided as a Source Data file.

With SC-CM treatment or Transwell co-culture with Schwann cells in vitro, CAFs expressed higher levels of inflammation-related genes (Fig. 6a and Supplementary Fig. S7d, e). Thereafter, the culture medium of SC/CAF alone/SC-induced CAFs was used to culture PDAC cells. The culture medium from Schwann cells or CAFs alone could enhance the migration abilities of PDAC cells (Fig. 7a and Supplementary Fig. S9a). More importantly, the culture medium of induced iCAFs by co-culture with Schwann cells or SC-CM facilitated PDAC cells to acquire strong capacities for migration (Fig. 7b–d and Supplementary Fig. S9b, c).

Using a patient-derived xenograft (PDX) mouse model, we found that with SC-CM treatment, patient-derived tumor tissues not only formed larger subcutaneous tumors in nude mice but also resulted in a higher incidence of tumor formation (25 vs. 16.7%, *n* = 12) (Supplementary Fig. S9d–g).

To assess the effect of neuro-stroma milieu on PDAC progression in vivo, PDAC cells mixed with SC-CM/IL-1RA-pretreated CAFs or control CAFs were injected into the pancreases (Fig. 7e). We found that SC-CM activated CAFs significantly increased PDAC progression in vivo (Fig. 7e–g and Supplementary Fig. S9h–l, G2 vs G1), and IL-1RA treatment substantially suppressed tumor sizes (Fig. 7e–g and Supplementary Fig. S9h–l, G3 vs G2).

To investigate this effect of SC-CM/activated CAF on tumor metastasis, PDAC cells pretreated by SC-CM/CAF-CM/inhibitors were injected into the spleen (Fig. 7h). Larger metastatic tumor and higher metastatic frequency were observed in the group with SC-CM or SC-induced CAF-CM treatment (Fig. 7i–l and Supplementary Fig. S9m, n, G2 vs G1; G5 vs G4; Supplementary Fig. S9o–s, IL-1α (G3) was used as a positive control). Consistently, iMDK could rescue the SC-CM-induced tumor metastasis, and IL-1RA could abolish malignant CAF phenotype induced by SC-CM (Fig. 7i–l and Supplementary Fig. S9m, n, G3 vs G2; G6 vs G5; Supplementary Fig. S9o–t, IL-1RA without SC-CM (G4) or CAF-CM (G5) were used as negative controls).

Collectively, we could also conclude that Schwann cells promote PDAC progression in vivo by shaping the phenotypes of tumor cells and CAF, which was induced by MDK and IL-1, respectively.

## Discussion

PDAC is characterized by extensive stroma and prominent neural alterations. The neural niche in the TME provides various cellular components and soluble molecules that facilitate cancer cell survival, proliferation, invasion, and motility^54^. Increasing evidence highlighted the critical role of nerve, especially Schwann cells, in PDAC development and progression^21,30^. The lack of a reliable modeling system has resulted in a paucity of studies on neural niches in the TME. Fortunately, a large number of PDAC tumor tissues, together with adjacent non-tumor tissues in our center (Ruijin Hospital, Shanghai Jiao Tong University School of Medicine, China), were used to investigate the clinical significance of Schwann cells in the TME. Similar to previous studies, we demonstrate that accumulation of Schwann cells in tumor tissues are associated with poor prognosis^9,30,55^.

Interestingly, Schwann cells are surrounded by stroma and tumor cells in the neuro-stroma niche of the TME (Fig. 1f). Schwann cells and other nerve cells are relatively rare in the TME compared with stromal and tumor cells; therefore, scRNA-seq and ST were applied to PDAC tissues with neural hypertrophy. Not surprisingly, only a very small population of Schwann-like cells cluster was detected in scRNA-seq, which was consistent with the previous findings^32,33^. However, scRNA-seq revealed the heterogeneity of TME cells in the context of neural hypertrophy, including basal-like and classical tumor cells, stellate-like cells, myCAF, and iCAFs. Basal-like subtype tumors not only exhibit faster growth rates but also enhanced migration and invasion capabilities compared to classical tumors. Moreover, basal-like subtype tumors have a poor prognosis^40,41,46,56^. As a type of pericyte primarily located at the base of the acini and around vascular, stellate-like cells in our study also express some markers of other mesenchymal cells, e.g., pericytes/vascular smooth muscle cells in breast cancer^57^. Currently, the origin of CAFs is still controversial. Although it was widely reported that pancreatic stellate cells (PSCs) were the major source of CAFs in PDAC^43^, Helms et al. reported that only 10–15% of CAFs originated from Fabp-GFP marker PSCs in mice model by transplanting tumor-stroma^58^. Cell fate trajectory analyses in our study suggested that stellate cells were precursors of the two main PDAC CAF subsets, myCAFs and iCAFs (Fig. 2j). Further experiments in molecular biology are required to validate the hypothesis. In PDAC, iCAFs and myCAFs coexist as mutually exclusive and reversible subtypes^52^. iCAFs are characterized by their cytokine-secreting properties. In tumor organoid-conditioned media, CAFs undergo a specific loss of myofibroblastic features but acquire phenotypes, including morphological activation, enhanced proliferation, and upregulated IL-6, IL-11, and leukemia inhibitory factor (LIF)^43^. iCAFs are potential mediators of immune suppression and play a role in promoting cancer progression^6,59–63^. scRNA-seq indicated that the nerve development and neural plasticity pathways were enriched in both basal-like tumor cells and iCAFs, suggesting an interplay between basal-like tumor cells, iCAFs, and nerves.

ST provides an unbiased and comprehensive picture of spatial composition to generate tissue atlases and have been adopted in a range of development, physiological and disease context, including cancers^64^. ST analysis indicates that iCAFs were co-localized with cancer cells expressing the stress-response gene module^65^. In our study, ST analysis showed that basal-like and iCAF-related signatures were enriched in the regions of Schwann cells, whereas the regions exhibiting classical- and myCAF-related signature were farther away from Schwann cells, suggesting that Schwann cells may induce the switch of tumor cells and CAFs to basal-like cells or iCAFs, respectively.

As the most prevalent cell type in peripheral nerves, Schwann cells are activated during tumor progression, even during the preneoplastic stage of PDAC^17^. They are detectable around PanIN lesions in genetically engineered PDAC mouse models^17^. During PDAC development and progression, Schwann cells work as paths of axonal guidance toward tumor cells, and lead to “neurogenesis” in TME, which is supported by histopathological manifestations with increased neural density and hypertrophy in tumor tissues^55,66^. Human and transgenic mouse PDAC harbor abundant Schwann cells, which are widely dispersed in the tumor-stroma^17^. The ST results indicated that the subtypes of tumor cells and CAFs adjacent to Schwann cells were distinct from those farther away. Schwann cells may contribute to the remodeling of the neuro-stroma niche in PDAC tissues.

Schwann cells have recently attracted extensive attention. Both co-culture and the conditioned medium of Schwann cells increase the migratory and invasive abilities of PDAC cells^27,29^. Moreover, Schwann cells enhance prostate and pancreatic cancer cell invasion via laminin-binding A6 integrin^67^ and promote EMT and invasion abilities of lung cancer cells through the CXCL5/CXCR2/PI3K/AKT/GSK-3beta/Snail-Twist pathway^26^. Consistent with these findings^26,27,29,67^, we also demonstrate that Schwann cells could promote the tumorigenicity of PDAC cells using in vivo and in vitro functional assays. More importantly, the co-culture of Schwann cells and CAFs can induce the switch of CAFs to pro-tumor iCAFs, which accelerates the progression of PDAC. The current study focuses on MDK and IL-1α as two mediators of Schwann cells in tumor cells and CAFs. Pharmacological inhibition of MDK and IL-1R antagonist could substantially abolish the Schwann cells induced PDAC progression in vivo. Other neurotrophic factors or chemokines may play similar roles in the neuro-stroma niche. However, the mechanism of the inclination of Schwann cells to migrate toward tumors and the effects on initiation of the neuro-stroma niche remain to be elucidated. As an important participant in tumor progression, IL-1α could also have multiple source and effector cells in TME^68^.

In summary, the current research reveals the heterogenicity of tumor cells and CAFs in the neuro-stroma niche by an integrated analysis of RNA sequencing at the single-cell level, spatial gene expression profiling, and histology. The study further demonstrates that Schwann cells can induce malignant progression of tumor cells and CAFs by releasing MDK and IL-1α. In the future, it will be important to explore the roles of more key factors, such as glial cell-derived neurotrophic factors, neural growth factors, and chemokines, in shaping the neuro-stroma niche. We hope that our study will provide a strong rationale for considering the effects of Schwann cells on shaping the main cells in the TME, and shed light on personalized therapy targeting Schwann cells.

## Methods

This research complies with all relevant ethical regulations. All experiments were reviewed and approved by Ruijin Hospital.

### Human tissue samples and tissue microarrays

Human pancreatic ductal adenocarcinoma (PDAC) resection specimens were obtained from patients who underwent pancreatectomy at Ruijin Hospital (Shanghai, China) between January 2016 and December 2018. The sample collection and preparation protocol were approved by the Ruijin Hospital Ethics Committee (reference number: 2013-70). All samples were obtained with written informed consent and were fully anonymized. Consent to publish relevant clinical information potentially identifying individuals (e.g., age, gender, histological grade, etc.) was obtained. This research was conducted according to the principles of the Declaration of Helsinki.

All patients were enrolled if: (1) the histopathological diagnosis with PDAC; (2) no prior anti-cancer treatments; (3) no other malignant history; (4) follow-up was completed within the scheduled time frame. Patients with infectious diseases, rheumatic diseases, or other malignancies were excluded. The samples were confirmed based on the two pathologists’ assessments. A total of 187 pairs of normal pancreases and PDAC were obtained from surgical specimens for tissue microarrays, including 112 males and 75 females with median ages of 62 and 67 years old.

### H&E, IHC, and IF

H&E, IHC, and IF staining were performed in PDAC tissues from archived formalin-fixed paraffin-embedded (FFPE) tumors and were performed according to standard protocols. For IHC, the slides were incubated with PGP9.5 (Servicebio, GB11159, 1:500), p75NRT (Abcam, ab52987, 1:100), GFAP (Proteintech, 16825, 1:200), S100β (Servicebio, GB11359, 1:150), IL-6 (Abcam, ab9324, 1:50), α-SMA (Abcam, ab5694, 1:200), N-Cadherin (Proteintech, 22018-1-AP, 1:100), Fibronectin (Proteintech, 15613-1-ap, 1:200), FAP (Cell signaling technology [CST], 66562,1:100) followed by HRP conjugated Goat Anti-Mouse IgG (H + L) (Servicebio, GB23301, 1:200) or HRP conjugated Goat Anti-Rabbit IgG (H + L) (Servicebio, GB23303, 1:200) and diaminobenzidine (Brown) (DAB, Dako). The slides were counterstained with hematoxylin (Dako), which stains nuclei blue, contrasting with the brown of HRP-DAB. Fibroblast morphology was confirmed based on the two pathologists’ assessments. IF was performed according to the manufacturer’s instructions (Panovue, 10004100100). Briefly, slides were incubated with primary antibodies for S100β (Servicebio, GB11359, 1:500), p75NRT (Abcam, ab52987, 1:100), α-SMA (Abcam, ab5694, 1:200), IL-6 (Abcam, ab233706, 1:50), and IL-1α (Proteintech, 16765-1-AP, 1:50) overnight at 4 °C, and stained with HRP-labeled goat anti-mouse/rabbit IgG secondary antibodies.

### scRNA-seq

The BD Rhapsody system was used to capture transcriptome information. Quantified by a High-Sensitivity DNA chip (Agilent) on a Bioanalyzer 2200 and Qubit High-Sensitivity DNA analysis (Thermo Fisher Scientific), sequencing was performed using HiSeqXten (Illumina, San Diego, CA, USA) with a 150 bp paired-end run. scRNA-seq was performed on single-cell suspensions with viability >70%. Single-cell capture was achieved by randomly distributing a single-cell suspension in >200,000 microwells using the limiting dilution method. To pair the beads with cells, beads with oligonucleotide barcodes were added to the saturation state in the microwell. Cells were lysed in the micropores, allowing polyadenylated RNA molecules to hybridize into the beads. The beads were collected and followed by reverse transcription and ExoI digestion. During cDNA synthesis, each cDNA molecule was marked with a unique molecular identifier (UMI) at the 5’ end (that is, the 3’ end of the mRNA transcript), and a cell barcode was used to mark its cellular origin. The BD Rhapsody Single Cell Whole Transcriptome Amplification (WTA) workflow was used to prepare the entire transcriptome library, which included random primer and extension (RPE), RPE amplification polymerase chain reaction (PCR), and WTA index PCR.

### scRNA-seq statistical analysis

Data analysis of scRNA-seq was performed by NovelBio Bio-Pharm Technology Co., Ltd. with the NovelBrain Cloud Analysis Platform. The adapter sequence was filtered using Fastp with the default parameter, and then low-quality reads are removed. To identify the cell barcode whitelist, we used UMI tools for analysis. Mapped to the human genome (Ensemble version 91) using STAR mapping with customized parameters from the UMI-tools standard pipeline, the UMI counts of each sample were obtained. Cells containing over 200 expressed genes and mitochondrial UMI rates below 40% passed the cell quality filtering, and mitochondria genes were removed. The Seurat package (version: 3.1.4, https://satijalab.org/seurat/) was used for cell normalization and regression based on the expression table according to the UMI counts of each sample and the percentage of mitochondria to obtain scaled data.

Utilizing the graph-based cluster method (resolution = 0.8), we acquired the unsupervised cell cluster result based on the principal component analysis (PCA) top ten principal components, and we calculated the marker genes using the FindAllMarkers function with the Wilcoxon rank-sum test algorithm under the following criteria:(1) lnFC >0.25; (2) *P* value <0.05; and (3) min.pct >0.1. To identify the cell type in detail, clusters of the same cell type were selected for re-t-SNE analysis, graph-based clustering, and marker analysis, and single-cell data were further processed using only clusters identified as malignant and fibroblasts. Re-clustering was performed on malignant cells and fibroblasts using a method similar to that described previously^69^.

GO and KEGG analysis was performed to elucidate the biological implications of marker genes and differentially expressed genes^70^. GO annotations were downloaded from NCBI (http://www.ncbi.nlm.nih.gov/), UniProt (http://www.uniprot.org/), GO (http://www.geneontology.org/), and KEGG (https://www.genome.jp/kegg/). Fisher’s exact test was applied to identify significant GO and KEGG categories, and FDR was used to correct *P* values.

CytoTRACE Analysis: We applied CytoTRACE Analysis for cell development analysis with default parameters, and the CytoTRACE score of each cell was calculated based on the expression matrix^39^.

TCGA Analysis: The same type of cancer data as clinical information can be downloaded from The Cancer Genome Atlas (http://cancergenome.nih.gov). The Kaplan–Meier test was used to analyze the gene set relationships. The threshold of significance was defined by the *P* value.

Pseudo-time analysis: We applied single-cell trajectory analysis utilizing Monocle2 (http://cole-trapnell-lab.github.io/monocle-release) using DDR-Tree and default parameters. Before Monocle analysis, we selected marker genes from the Seurat clustering results and raw expression counts of the filtered cells. Based on pseudotime analysis, branch expression analysis modeling (BEAM analysis) was applied for branch fate-determined gene analysis.

### Microarray-based ST

Tissue permeabilization and spatial transcriptomic sequencing were performed using Visium Spatial Gene Expression Slides & Reagent Kits. Quantified by the qubit high-sensitivity DNA assay (Thermo Fisher Scientific), the size distribution of the final libraries was further determined using a high-sensitivity DNA chip on a Bioanalyzer 2200 (Agilent). All libraries were sequenced using an Illumina sequencer (Illumina) with a 150 bp paired-end run. After surgically removed, PDAC tumor tissues were embedded in the optimal cutting temperature (OCT) compound (Sakura). Further, the tissues with OCT compound were immediately frozen using dry ice. Tissue sections (10 μm thick) were placed within the frames of capture areas on Visium Spatial slides (10X Genomics) and imaged using a Pannoramic MIDI microscope (3DHISTECH) after H&E staining. To optimize permeabilization time, we performed pre-permeabilization using Visium spatial tissue optimization slides and reagent kits (10X Genomics). For tissue permeabilization and spatial transcriptomic sequencing, we first performed the reverse transcription by incubating the stained slides in RT Master Mix at 53 °C for 45 min after permeabilization. To initiate second strand synthesis, the tissue sections were incubated for 15 min at 65 °C with a Second Strand mix added to the slides. After transferring the cDNA, it was purified via the barcode cDNA and amplified. The amplified barcoded cDNA was fragmented, A-tailed, ligated with adapters, and amplified using PCR.

### ST statistical analysis

We applied fastp with default parameter filtering of the adapter sequence and removed low-quality reads to achieve clean data^71^. Then, feature-barcode matrices were obtained by aligning reads to the human genome (GRCh38) using SpaceRanger v1.1.0. To minimize the sample batch, we applied downsample analysis among samples sequenced according to the mapped barcoded reads per spot of each sample, and finally achieved the aggregated matrix.

The Seurat package (version 3.2, https://satijalab.org/seurat/) was used for spot normalization and regression. The fastMNN function from the R package scran (v1.10.2) was used to apply the mutual nearest neighbor method to correct for batch effects among samples^72^. PCA was constructed based on scaled data with all highly variable genes, and the top 30 principals were used for UMAP construction. Utilizing the graph-based cluster method, we acquired the unsupervised cell cluster result based on the PCA top 30 principal components, and we calculated the marker genes using the FindAllMarkers function with the Wilcoxon rank-sum test algorithm under the following criteria:(1) lnFC >0.25; (2) *P* value <0.05; and (3) min.pct >0.1. Spatial feature expression plots were generated using the SpatialFeaturePlot function in Seurat (version 3.1.3) and STUtility R package (version 1.0.0).

ssGSEA: We applied gene set enrichment analysis based on single cell-specific gene sets and normalized gene expression matrix by ssGSEA function in the GSVA package to achieve the gene enrichment score of each spot^73^.

GO analysis was performed similarly to scRNA-seq.

QuSAGE Analysis: To characterize the relative activation of a given gene set, such as pathway activation, we performed a QuSAGE (2.16.1) analysis^45^.

### Cell culture

All cell lines were maintained at 37 °C with 5% CO_2_. Panc-1, RSC96, sNF96.2, CAFs, hPSCs, and NF cells were cultured in DMEM (Meilunbio, China) containing 10% fetal bovine serum (FBS) (Gibco; Life Technology) and 50 μg/mL penicillin/streptomycin (P/S). CFPAC-1 cells were cultured in IMDM (BIOAGRIO) containing 10%FBS and 50 μg/mL P/S. Panc-1 (CRL-1469™), CFPAC-1 (CRL-1918™), RSC96 (CRL-2765™), and sNF96.2(CRL-2884™) were purchased from ATCC. NF and CAFs were isolated from human normal/tumor tissues. hPSC was a gift from Dr. Yuan Fang, which were purchased from ScienCell Research Laboratories, Carlsbad, CA (the HPaSteC cells, #3830). All cells were checked routinely for the absence of mycoplasma contamination. Short tandem repeat profiling was used to authenticate all the cell lines.

### Fibroblasts isolation and analysis

NF and CAFs were isolated from human normal pancreases (NF)/PDAC tissues (CAFs) as previously described in ref. ^74^ with some modifications. Briefly, the samples were cut into small pieces and collagenase was digested. The tissue was seeded in the culture dish with 10% FBS/DMEM. After 7–10 days, fibroblasts started to grow out of the tissue into the dish. Fibroblasts were confirmed by morphology and expression of CAF markers, including α-SMA (Abcam, ab5694, 1:1000 and 1:200) and FAP (CST, 66562, 1:1000), by Western blotting and IF.

### CM concentration

RSC96 and sNF96.2 cells were cultured in 10% FBS/DMEM until reaching 80–90% confluence. Thereafter, the medium was replaced with DMEM without FBS or phenol red and cultured for 24 h. Then, the cell culture medium was harvested and concentrated using an Amicon Ultra-4 centrifugal filter device (3000 MWCO cutoff) (Millipore) at 3000×*g* for 1 h. Protein concentrations were determined using the bicinchoninic acid assay (Thermo Fisher Scientific). The concentrated CM was collected and stored at −80 °C until further use.

### Co-culture of Schwann cells, cancer cells, and CAFs in vitro

For incubation of tumor cells with SC-CM, cancer cells were incubated with 100 µg/ml SC-CM for 72 h.

For direct co-culture, 1 $\times$ 10^5^ CAFs and 1 $\times$ 10^5^ RSC96 cells were mixed and seeded in six-well plates overnight. About 2 $\times$ 10^5^ CAFs or 2 $\times$ 10^5^ RSC96 cells were cultured alone as controls. The cells were cultured in 10%FBS/DMEM for 24–48 h, and the supernatant was harvested using a 0.22 µm filter. For Transwell-based co-cultures, 2 $\times$ 10^5^ CAFs were seeded in the lower compartment of six-well plates and 2 $\times$ 10^5^ RSC96 were seeded on top of the Transwell membrane (Corning, 0.4 µm). After 48 h of co-culture, CAFs were washed by PBS three times, and then an equal number of CAFs were cultured individually in fresh medium for 24–48 h, and the supernatant was harvested using a 0.22 µm filter. For the incubation of CAFs with SC-CM, CAFs were incubated with 20–100 µg/ml SC-CM for 48 h. After being rinsed by PBS for three times, an equal number of CAFs were cultured in fresh medium for 24–48 h, and CAFs supernatant was collected. CAFs CM were incubated with 1–10 $\times$ 10^5^ Panc-1 or CFPAC-1 for an additional 48 h. Then cancer cells were harvested for further study.

### Ex vivo tissue culture

An ex vivo culture model of tumor tissue was established, as previously described in ref. ^51^ with some modifications. Briefly, fresh PDAC tissues were dissected into approximately 1 mm^3^ pieces and then submerged in 10% FBS/DMEM with 100 µg/ml RSC96 CM for 24‒48 h.

### PDAC liver metastatic, orthotopic, and PDX models

All experiments performed on mice were reviewed and approved by the review board on the use of living animals at Ruijin Hospital. Nude BALB/c mice (6 weeks old) were purchased from the Chinese Academy of Sciences (Shanghai, China) and maintained in a specific pathogen-free facility. Panc-1 cells were labeled with firefly luciferase using an in vivo imaging system (IVIS). Luciferin emission imaging of isoflurane-anesthetized animals was performed using the IVIS Spectrum (Tanon) after intraperitoneal injection of d-luciferin (150 mg/kg; Promega, P1043) into mice. Tumor size was measured using a digital caliper, and tumor volume was calculated as 0.5 × length × width^2^. No sex selection was performed in this study. Mice were euthanized before the tumor volume exceeded 1500 mm^3^.

The orthotopic implantation model was established as previously reported^75^. Briefly, for the co-injection model in Fig. 6n, 2 × 10^6^ luciferase-expressing Panc-1, 2 × 10^6^ CAF-2 and 1 × 10^5^ sNF96.2 cells were suspended in 25 μL PBS and injected into the body of the pancreas. After 14 days, mice were divided into control or inhibitors groups, the latter were treated with iMDK (9 mg/kg, MCE, HY-110171A) and IL-1RA (25 mg/kg, MCE, AMG-719) by intraperitoneal injection every 48 h.

For the co-injection model in Fig. 7e, 2 × 10^6^ Panc-1 or CFPAC-1 and 2 × 10^6^ CAF-2 (w/ or w/o RSC96 CM/IL-1RA (1 μg/ml) treatment) were injected into the pancreas.

For the liver metastatic models in Fig. 7h, cells suspended in 30 μL PBS were injected into the spleen. About 2 × 10^6^ Panc-1 or CFPAC-1 cells were treated with ctrl CM(G1), SC-CM(G2), SC-CM+iMDK (2.5 μM) (G3), normal CAF-CM(G4), SC-induced CAF-CM(G5), or SC + IL-1RA (1 μg/ml) induced CAF-CM (G6) for 72 h before injection. For Sup Fig. 9o, 2 × 10^6^ CFPAC-1 cells were treated with ctrl CM (G1), normal CAF-CM (G2), recombined IL-1α-induced (10 ng/ml) CAF-CM (G3), IL-1RA (1 μg/ml) treated CAF-CM (G4) or IL-1RA (1 μg/ml) only (G5) for 72 h before injection.

For the PDX model, fresh PDAC tissues were dissected and washed twice with PBS, trimmed into 1 mm^3^ fragments, and finally submerged in 10% FBS/DMEM with or without RSC96 CM (100 μg/ml) for 48 h. After ex vivo culture, the tumor samples with Matrigel (Corning) were subcutaneously transplanted into the dorsal flank of mice.

### Bulk RNA sequencing

CFPAC-1, CAF-1, and CAF-2 were lysed with TRIzol (Invitrogen, 15596026), and total RNA was extracted using RNeasy Mini Kit (Qiagen, 74104), according to the manufacturer’s instructions. RNA sequencing libraries were generated using the KAPA RNA library preparation kit (Illumina) and the KAPA dual-indexed adapter kit (Illumina), and sequenced on an Illumina HiSeq X10 platform (2 × 150 bp).

### Mass spectrometry (MS)

MS was performed to validate candidate molecules in the CM of Schwann cells. Briefly, RSC96 and sNF96.2 CM were denatured, alkylated, and digested in situ with trypsin. Tryptic peptides were analyzed using an Orbitrap Fusion LUMOS mass spectrometer (Thermo Fisher Scientific) coupled to an Easy-nLC 1200 via an Easy Spray (Thermo Fisher Scientific).

### Cell proliferation assays

Cell Counting Kit-8 (CCK-8) was used to estimate the cell proliferation rate according to the manufacturer’s instructions (Meilunbio, MA0218). Briefly, Panc-1 and CFPAC-1 cells were plated in 96-well plates at a density of 1–5 × 10^3^ cells/well and the optical density at 450 nm (OD450) was measured (Biotek, USA) every 24 or 48 h for 5 days. For colony formation assays, 1 × 10^3^ cells were seeded in six-well plates for 10 days, and surviving colonies were stained and counted using 1% crystal violet (Beyotime, C0121). EdU cell proliferation staining was performed using an EdU kit (BeyoClick™ EdU Cell Proliferation Kit with Alexa Fluor 555; Beyotime, C10338) according to the manufacturer’s instructions. Panc-1 (1 × 10^6^) or CFPAC-1 cells (3 × 10^5^) were seeded in six-well plates overnight and were incubated with EdU for 2 h.

### Transwell migration and invasion assays

Panc-1 (1–5 × 10^4^) and CFPAC-1 (1 × 10^5^) were seeded into Transwell inserts (8-μm pore size, BD Falcon) for migration assays. For the invasion assay, inserts were pre-coated with Matrigel (Corning, 354234), according to the manufacturer’s instructions. After 24 h of culture for the migration assays or 48 h for the invasion assays, the wells were stained with crystal violet (Beyotime, C0121). Migrated cells number was quantified in an automated fashion using the ImageJ v1.53e software.

### Cell cycle and apoptosis assays

For the cell cycle assay, 1 × 10^6^ cells were collected and fixed in 75% ethanol at 4 °C overnight. DNA was stained using propidium iodide (PI)/RNase Staining Buffer (BD Biosciences, USA), according to the manufacturer’s instructions. For the apoptotic assay, 1 × 10^6^ cells were incubated with 5 μl of FITC-conjugated annexin V (BD Biosciences, 556419) and 5 μl of PI (BD Biosciences, 550825) for 15 min at room temperature in the dark. Flow cytometry was performed using FACS (Becton-Dickinson, Bedford, MA, USA), and the results were analyzed using the FlowJo V10 software.

### Enzyme-linked immunosorbent assay (ELISA)

The levels of IL-1α and IL-6 in the CM samples from CAFs and Schwann cells were quantified using a commercial ELISA kit (MultiSciences, EK101A, EK301A, and EK106), according to the manufacturer’s instructions.

### Protein extraction and western blot assay

Total protein was extracted from cell pellets lysed with RIPA buffer (Sigma-Aldrich, R0278) containing 5 mM EDTA, 1 × Halt phosphatase inhibitor cocktail (Thermo Fisher Scientific, 78420), and 1 × Halt protease inhibitor cocktail (Thermo Fisher Scientific, 78429). For western blotting, an estimated 10–40 µg protein was loaded per well on a 10% SDS-PAGE gel and transferred onto a PVDF membrane (Thermo Fisher Scientific) and incubated with antibodies against α-SMA (Abcam, ab5694, 1:1000), FAP (CST, 66562, 1:1000), GAPDH (Santa Cruz Biotechnology, sc-47724, 1:1000), E-cadherin (CST, 3195, 1:1000), N-cadherin (CST,13116, 1:1000), Vimentin (CST, 5741, 1:2000), Fibronectin (CST, 26836, 1:2000), and β-catenin (CST, 8480, 1:1000) after blocking.

### Recombined protein and protein neutralization

For recombined protein, 10 ng/ml recombined human MDK (PeproTech, 450-16) were incubated with Panc-1 cells with for 24–48 h. About 1 ng/ml recombined human IL-1α (R&D systems, 200-LA-002/CF) were incubated with CAFs for 24 h for in vitro assays or 10 ng/ml for 72 h for in vivo assay. For protein neutralization, CM of Schwann cells were neutralized with 10 μg/mL anti-Midkine (Santa Cruz Biotechnology, sc-46701) and IL-1α (Proteintech, 16765-1-AP) for 1 h at 37 °C. Normal rabbit IgG (10 μg/mL, CST, 2729) alone was used as the control.

### RNA extraction, cDNA synthesis, and real-time PCR (qPCR)

Total RNA was extracted using TRIzol Reagent (Life Technologies, 15596026), and reverse transcription was performed using HiScript® Reverse Transcriptase (Vazyme, China, R101-02), according to the manufacturer’s instructions. For qPCR analysis, double-stranded cDNA was amplified using an SYBR Green PCR Kit (Vazyme, Q712) and detected using qTOWER384G (Analytikjena). The primer sequences used in this study are listed in Supplementary Table S2.

### Statistical analysis

Statistical analyses were performed using GraphPad Prism version 8.0. IHC/IF positive area and intensity were calculated by QuPath Version 0.1.2 (https://qupath.github.io/). Patients in the tissue microarray were divided into low and high SC area/iCAFs number groups according to the optimal cut-off value calculated using the X-Tile software^76^. K–M curve and Cox regression analysis was performed to assess the association with overall survival using SPSS v23 (IBM Inc.). All statistical tests were indicated in the figure legends.

### Reporting summary

Further information on research design is available in the Nature Portfolio Reporting Summary linked to this article.

## Supplementary information

Supplementary Information

Description of Additional Supplementary Files

Supplementary Data 1

Supplementary Data 2

Supplementary Data 3

Reporting Summary

## Data Availability

Bulk RNA sequencing data generated in this study are deposited in Gene Expression Omnibus under accession code GSE201601. scRNA-seq data generated in this study are deposited in Gene Expression Omnibus under accession code GSE202742. ST data generated in this study are deposited in Gene Expression Omnibus under accession code GSE202740. The mass spectrometry proteomics data have been deposited to the ProteomeXchange Consortium via the PRIDE^77^ partner repository with the dataset identifier PXD042556. The survival analyses and GSEA analyses in TCGA were derived from TCGA Research Network (http://cancergenome.nih.gov/). The remaining data are available within the Article, Supplementary Information or Source Data file. Source data are provided with this paper.

## Source data

Source Data
