# Supplementary material for: Schwann cells regulate tumor cells and cancer-associated fibroblasts in the pancreatic ductal adenocarcinoma microenvironment
Source: Nat Commun. 2023 Jul 31;14:4600. doi: 10.1038/s41467-023-40314-w (PMC10390497; doi:10.1038/s41467-023-40314-w)
Supplement: Supplementary file 1 — Supplementary Information [file 41467_2023_40314_MOESM1_ESM.docx]

**SUPPLEMENTARY INFORMATION**

**Schwann Cells Regulate Tumor Cells and Cancer-Associated Fibroblasts in the Pancreatic Ductal Adenocarcinoma Microenvironment**

Meilin Xue^1,2,3,4,#^, Youwei Zhu^1,2,3,#^, Yongsheng Jiang^1,2,3,#^, Lijie Han^1,2,3,#^, Minmin Shi^1,2,3^, Rui Su^4^, Liwen Wang^1,2^, Cheng Xiong^1,2,3^, Chaofu Wang^5^, Ting Wang^5^, Shijie Deng^5^, Dong Wu^4^, Yizhi Cao^1,2,3^, Lei Dong^5^, Fan Bai^6^, Shulin Zhao^1,2^, Xiaxing Deng^1,2^, Chenghong Peng^1,2^, Hongwei Li^1,2^, Jianjun Chen^4^, Baiyong Shen^1,2,3,*^, Lingxi Jiang^1,2,*^, Hao Chen^1,2,3,*^

^1^Department of General Surgery, Pancreatic Disease Center, Ruijin Hospital, Shanghai Jiao Tong University School of Medicine, Shanghai, China.

^2^Research Institute of Pancreatic Diseases, Shanghai Jiao Tong University School of Medicine, Shanghai, China.

^3^State Key Laboratory of Oncogenes and Related Genes, Institute of Translational Medicine, Shanghai Jiao Tong University, Shanghai, China.

^4^Department of Systems Biology, Beckman Research Institute of City of Hope, Monrovia, CA 91016, USA.

^5^Department of Pathology, Ruijin Hospital, Shanghai Jiao Tong University School of Medicine, Shanghai, China.

^6^Biomedical Pioneering Innovation Center (BIOPIC), School of Life Sciences, Peking University, Beijing, China.

^#^ These authors contributed equally

^*^ These authors jointly supervised this work. Correspondence: [shenby@shsmu.edu.cn](mailto:shenby@shsmu.edu.cn) (B.S.); [jlx12120@rjh.com.cn](mailto:jlx12120@rjh.com.cn%20(L.J) (L.J.); [haochendr@126.com](mailto:haochendr@126.com) (H. C.)

**Supplementary Figures 1-10**

**Supplementary Tables 1-2**


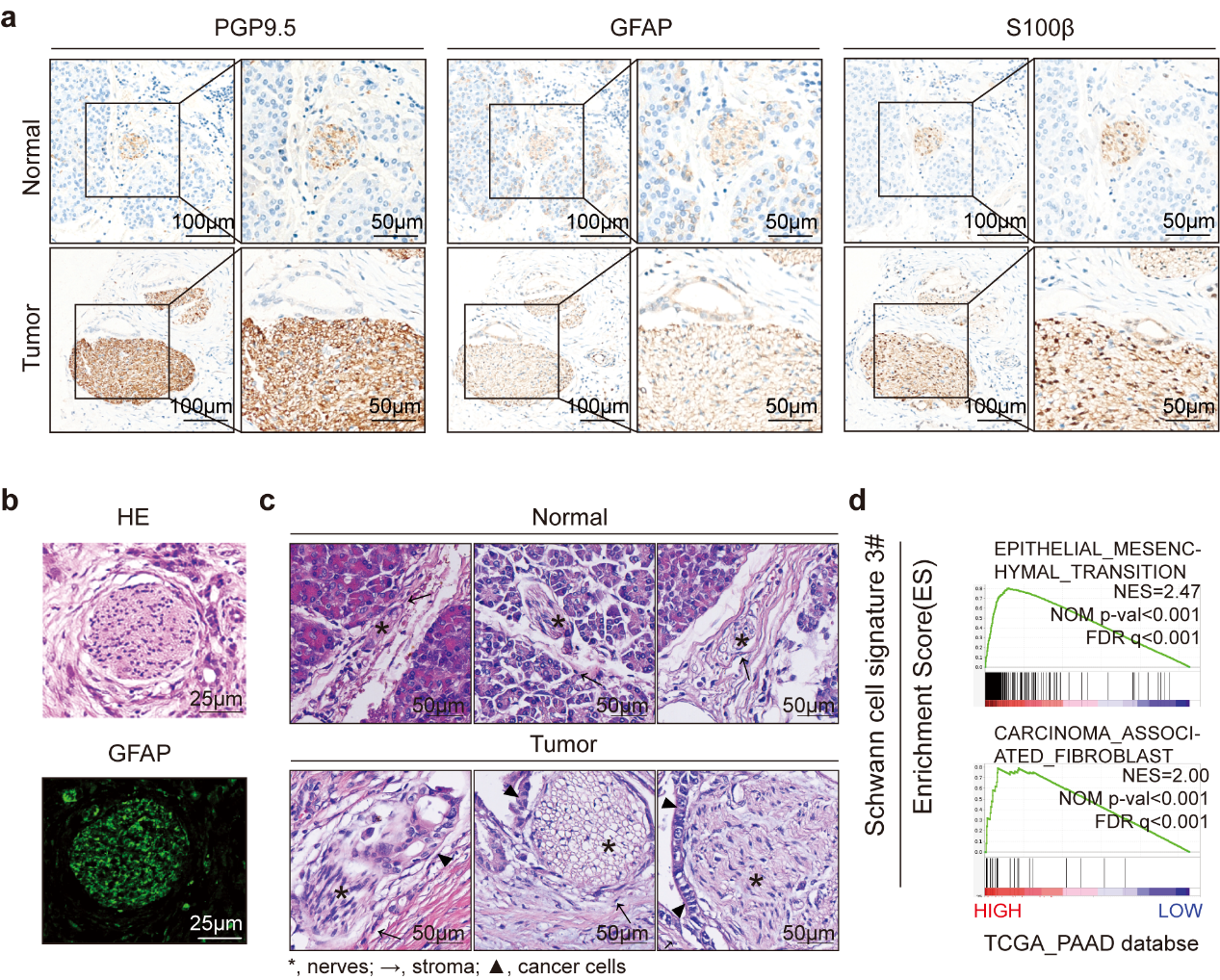


**Supplementary Figure S1. Prevalence of Schwann Cells Accumulation in the TME and its Clinical Significance**

**a.** Representatives of IHC staining images with anti-PGP9.5, GFAP, and S100β. Images are representative of 187 biologically independent PDAC samples with similar results. Scale bar, left, 100 µm; right, 50 µm.

**b.** The co-localization of nerve and Schwann cells shown by H&E and IF staining with anti-GFAP in PDAC tissue. Scale bar, 25 µm.

**c.** H&E staining showed nerves, stroma, and cancer cells in PDAC tumor and normal tissue. asterisk, nerves; arrow, stroma; triangle, cancer cells. Scale bar, 50 µm. b, c, Images are representative of 3 biologically independent PDAC samples with similar results.

**d.** GSEA plot showing that EMT and carcinoma-associated fibroblasts were enriched in PDAC samples with higher expression of Schwann cell signature. Pathway enrichment analysis was performed using data from the TCGA-PAAD cohort. NES normalized enrichment score, corrected for multiple comparisons using FDR method, *P*-value were showed in plots.


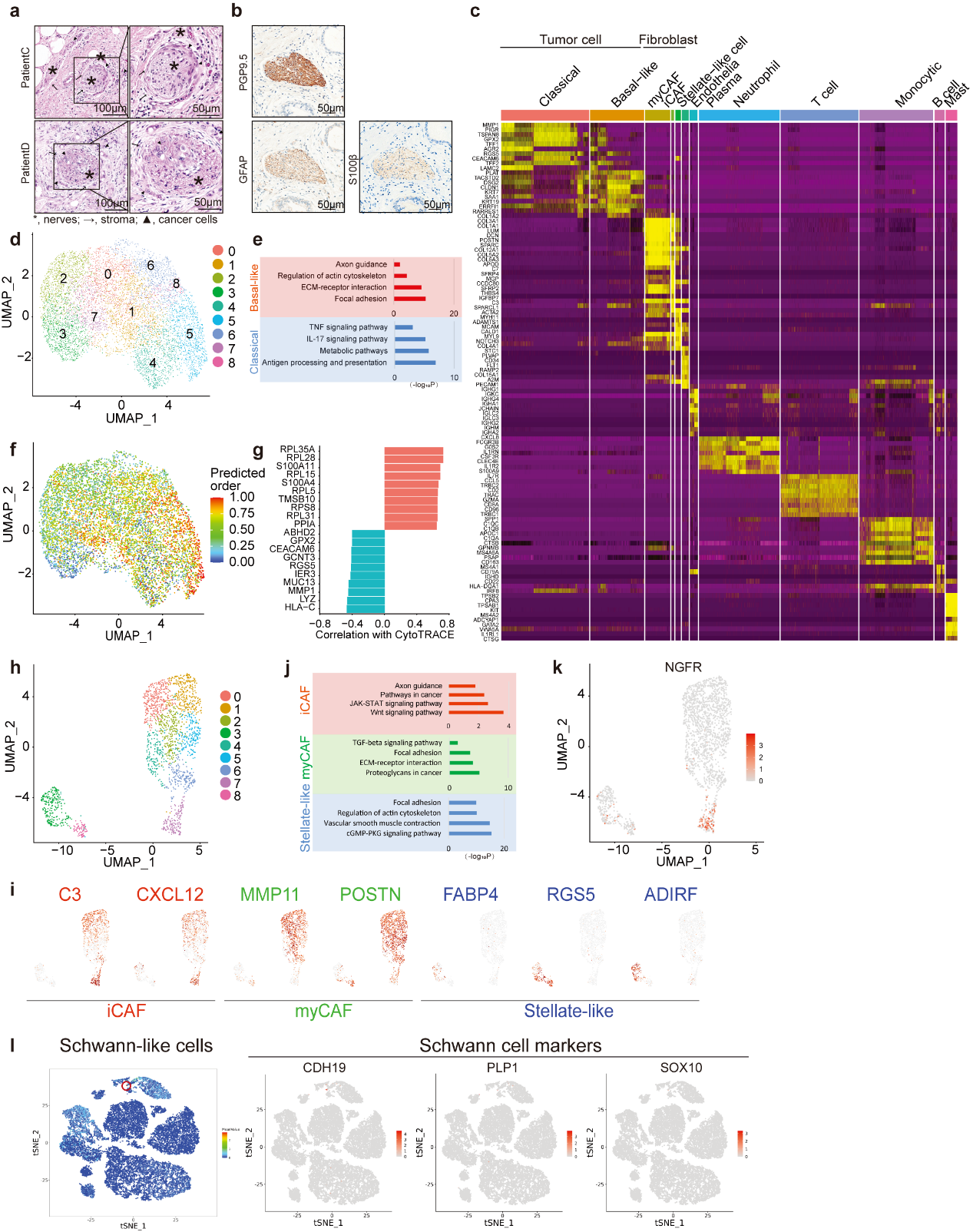


**Supplementary Figure S2. Single-Cell Analysis Reveals the Heterogeneity of Tumor Cells and CAFs in PDAC with Schwann Cells Accumulation**

**a.** Representative H&E images of PDAC tissues with neural hypertrophy. asterisk, nerves; arrow, stroma; triangle, cancer cells. Scale bar, left, 100 µm; right, 50 µm.

**b.** Representatives images of IHC staining with anti-PGP9.5, GFAP, and S100β for PDAC tumor from Patient B. Scale bar, 50 µm. a-b, Images are representative of 4 biologically independent PDAC samples with similar results.

**c.** Heatmap showing the expression of marker genes of each cell cluster in single cell RNA sequencing of four PDAC tissues.

**d.** UMAP plot showing nine different subclusters of cancer cells from four PDAC tissues.

**e.** KEGG analysis showing the upregulated pathways in basal-like and classical cancer cells.

**f, g.** CytoTRACE predicts the order (**f**) and significant genes (**g**) in cancer cells.

**h.** UMAP plot showing nine different subclusters of fibroblasts from PDAC tissues.

**i.** UMAP plot showing the marker genes of each fibroblasts cluster.

**j.** KEGG analysis showing the upregulated pathways in iCAFs, myCAFs, and stellate-like cells.

**k.** The relative expression level of *NGFR* in iCAFs.

**l.** *t*-SNE plot showing putative Schwann cells cluster identified by previously reported markers.

Statistical analysis: one-sided Fisher’s exact test (e, j).


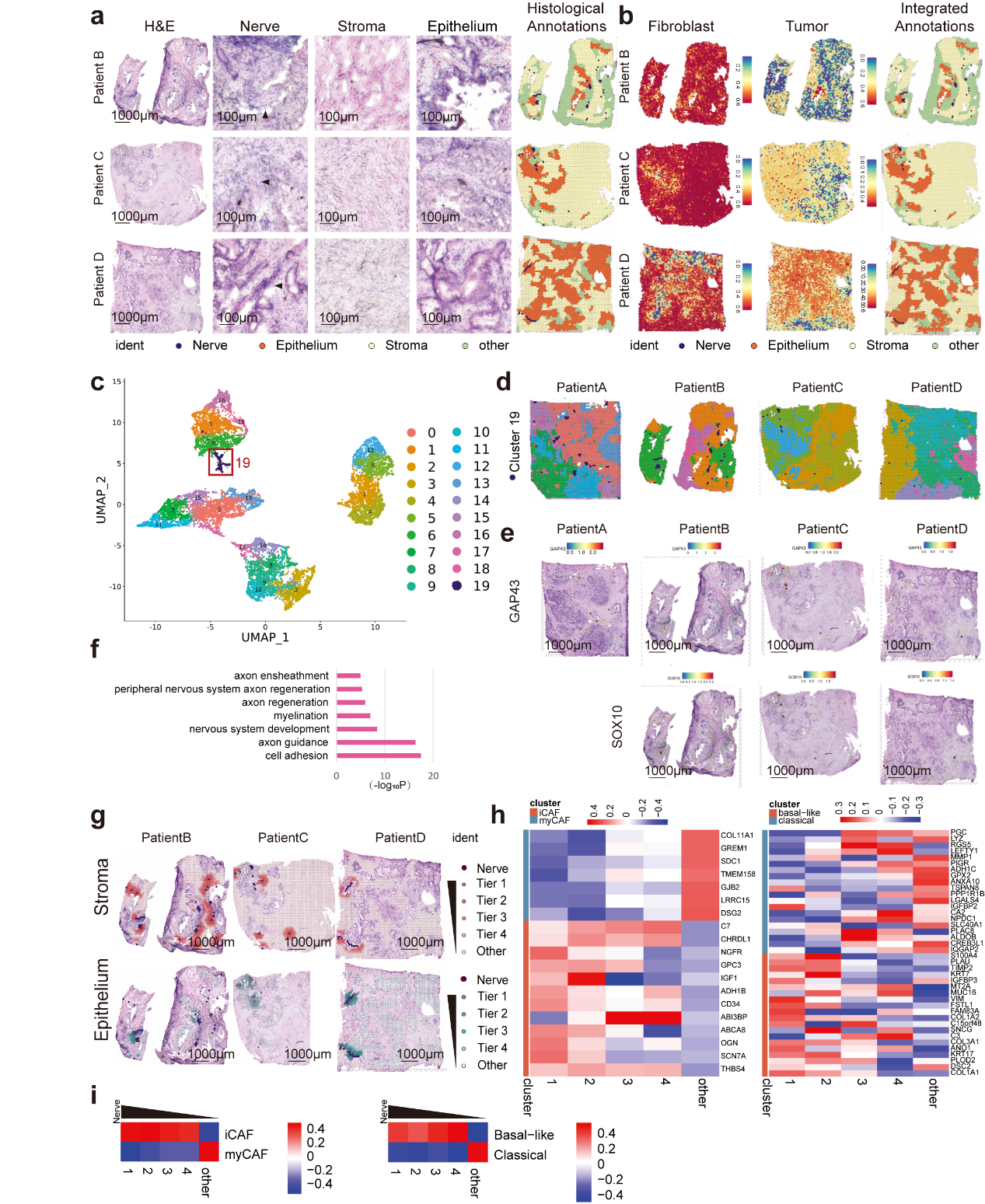


**Supplementary Figure S3. Microarray-Based Spatial Transcriptomics Reveals that Schwann Cells are Surrounded by iCAFs and Basal-Like Tumor Cells**

**a.** Histological annotations of tumor cryosection from Patients B‒D. Images are representative of 4 biologically independent PDAC samples with similar results. Scale bar, left, 1000 µm; right, 100µm.

**b.** Stroma and epithelium area were defined by integrating the results of ssGSEA and histologic annotation.

**c.** UMAP plot showing 20 different subclusters from four ST slides.

**d.** Subcluster 19 was co-localized with nerve region indicated by histological annotation in Patients A-D.

**e.** Schwann cell markers SOX10 and GAP43 were co-localized with nerve region in Patients B-D. Scale bar, 1000 µm.

**f.** GO analysis showing the enrichment of Schwann cell-related pathways in subcluster 19.

**g.** Perineural tier 1‒4 and other regions were defined according to the distance to nerve in the neuro-stroma niche of Patients B‒D. Scale bar, 1000 µm.

**h.** Heatmap showing the marker genes in iCAF, myCAF, basal-like and classical used for QuSAGE.

**i.** QuSAGE scores of iCAF, myCAF, basal-like and classical signatures in perineural tier 1-4 and other regions in the neuro-stroma niche. These signatures were defined by previously reported markers of iCAF, myCAF, basal-like and classical tumor cells.

Statistical analysis: one-sided Fisher’s exact test (f).


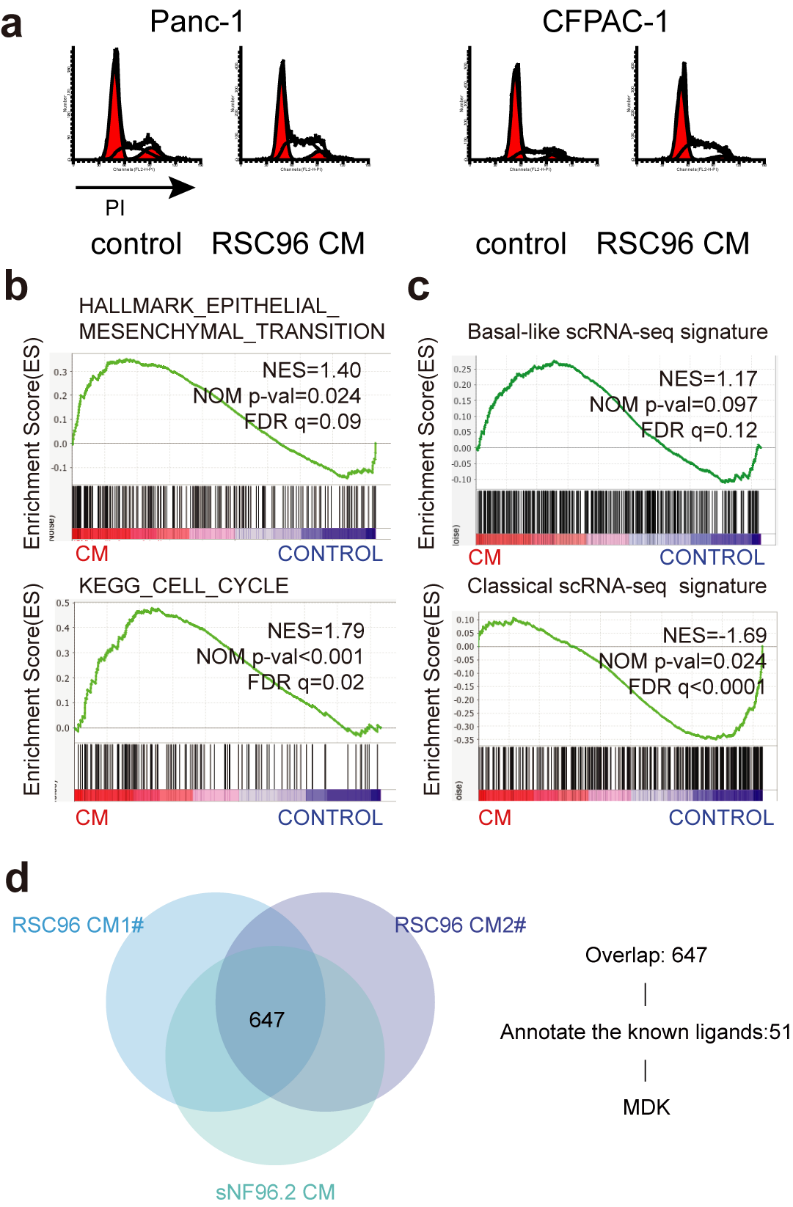


**Supplementary Figure S4. Schwann Cells Promote Malignant Progression in PDAC via MDK**

**a.** FACS plots depicting the cell cycle of Panc-1 and CFPAC-1 cells cultured with or without RSC96 CM.

**b, c.** GSEA showing the enrichment score of EMT and Cell cycle pathways (**b**) and the corresponding signatures of scRNA-seq (**c**) in cancer cells. Pathway enrichment analysis was performed based on bulk RNA-seq of CFPAC-1 cultured in RSC96 CM and control medium. NES normalized enrichment score, corrected for multiple comparisons using FDR method, *P*-value were showed in plots.

**d.** Schematic diagram of MDK selection from the MS data. #1 and #2 represent two biological replicates of the RSC96 CM.

*
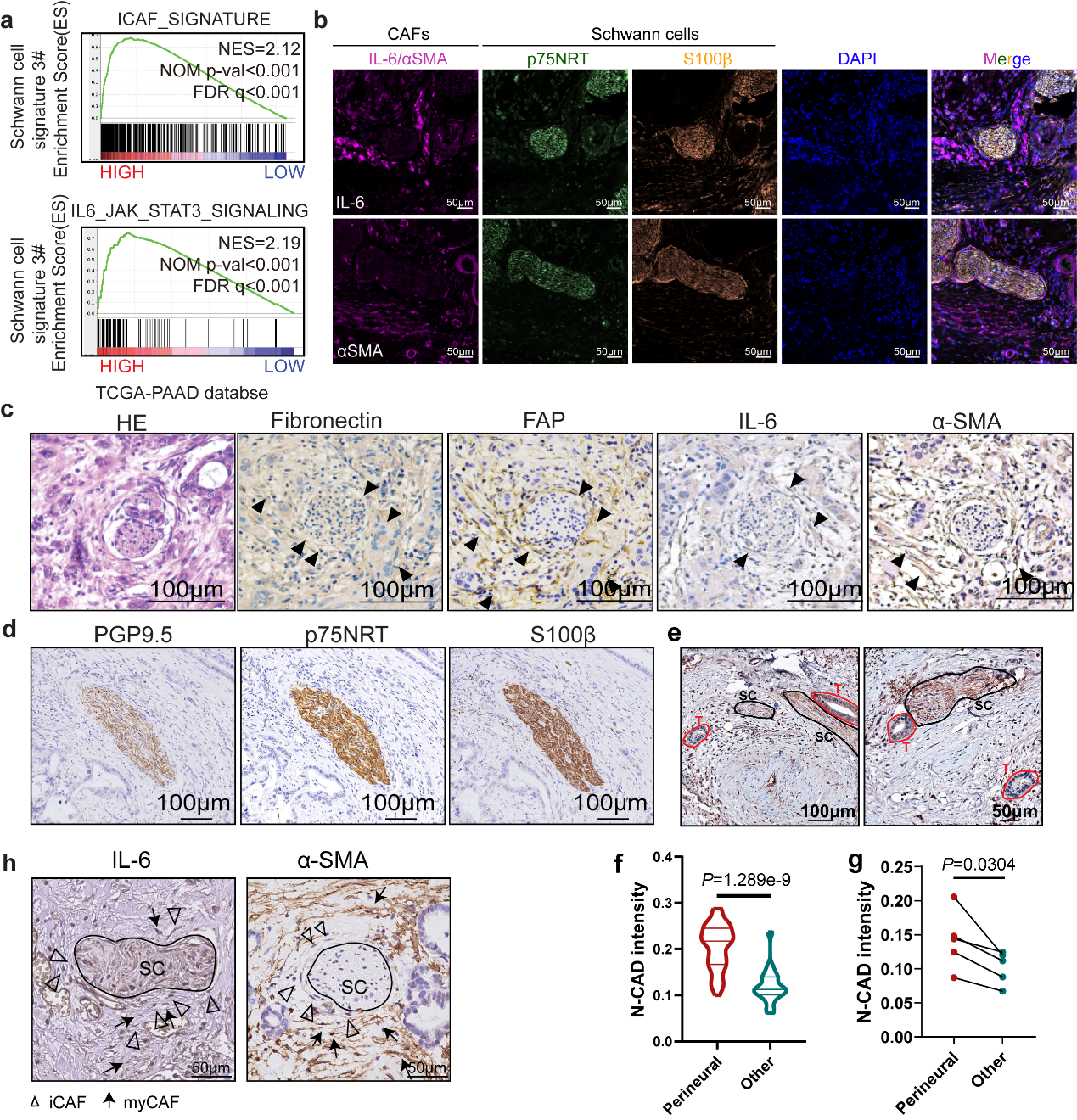
*

**Supplementary Figure S5. Perineural iCAFs widely exist in PDAC.**

**a.** GSEA plot showing that iCAF signature and IL6_JAK_STAT3 pathway were enriched in PDAC samples with high expression level of Schwann cell signature. Pathway enrichment analysis was performed using RNA-seq from the TCGA-PAAD database. NES normalized enrichment score, corrected for multiple comparisons using FDR method, *P*-value were showed in plots.

**b.** Representative images of IF with anti-IL-6 (marker for iCAFs) or anti-α-SMA (marker for myCAFs), and S100β/p75NRT (marker for Schwann cells) in the neuro-stroma niche. Scale bar, 50µm.

**c.** Representative images of IHC with anti-Fibronectin (marker for fibroblasts), FAP (marker for CAFs), IL-6 (marker for iCAFs) and α-SMA (marker for myCAFs) in the neuro-stroma niche. Scale bar, 100µm.

**d.** Representatives of IHC staining images with anti-PGP9.5, p75NRT, and S100β. Scale bar, 100µm. c, d. Images are representative of 3 biologically independent PDAC samples with similar results.

**e.** Representative images of IHC staining with N-Cadherin in human PDAC. SC, Schwann cells; T, tumor. Scale bar, 100µm. b, e, Images are representative of 5 biologically independent PDAC samples with similar results.

**f-g.** Quantification of N-Cadherin intensity in tumor cells in perineural or other regions in representative panel e left (**f**) or mean intensity of 5 independent PDAC patients **(g)**. For each violin, the minimum, first quartile, median, third quartile, and maximum were displayed.

**h.** Representative images of IHC staining with anti-IL-6 and α-SMA in human PDAC. Images are representative of 31 biologically independent PDAC samples with similar results. Scale bar, 50µm.

Statistical analysis: unpaired two-sided *t*-test (f); paired two-sided *t*-test (g).


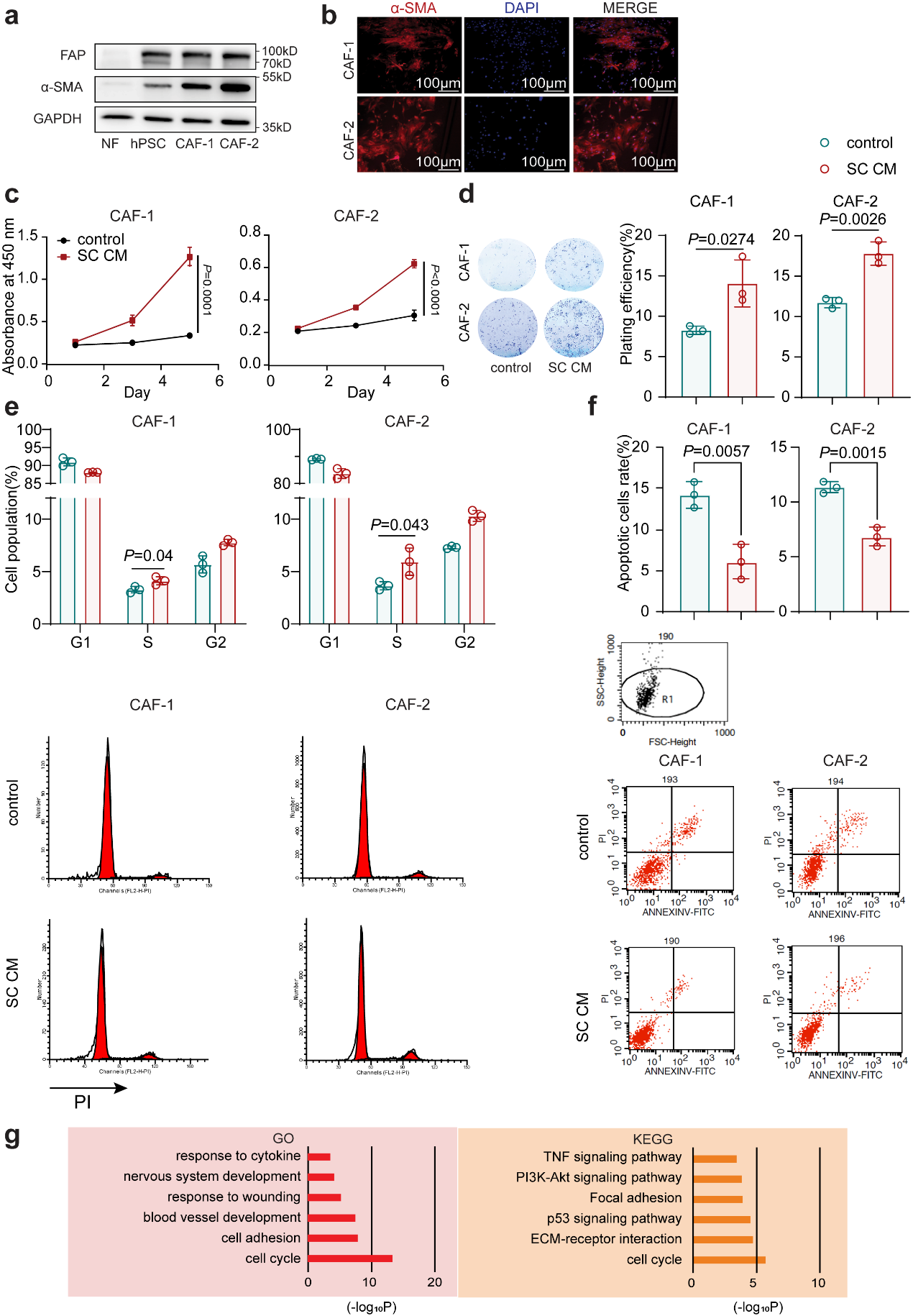


**Supplementary Figure S6. Schwann Cells Induce CAFs Proliferation**

**a.** The protein levels of FAP and α-SMA expression in hPSC and NF (normal fibroblast), CAF-1 and CAF-2 derived from PDAC tissues. Data are representative of n = 3 independent experiments.

**b.** Representative IF images with anti-α-SMA in CAFs. Scale bar, 100µm. Images are representative of 3 biologically independent experiments with similar results.

**c, d.** Effects of RSC96 CM on the proliferation of CAFs were evaluated by the CCK-8 assay (**c**) and colony formation assays (**d**).

**e, f.** Effects of RSC96 CM on cell cycle (**e**) and apoptosis (**f**) of CAFs were evaluated by flow cytometry. Gating strategies were shown in the bottom.

**g.** GO and KEGG analyses showing the enriched pathways in CAFs cultured with RSC96 CM.

c-f, Data are the mean ± s.d. of n = 3 independent experiments. Statistical analysis: two-way ANOVA (c); unpaired two-sided *t*-test (d-f); one-sided Fisher’s exact test (g). Source data are provided as a Source Data file.


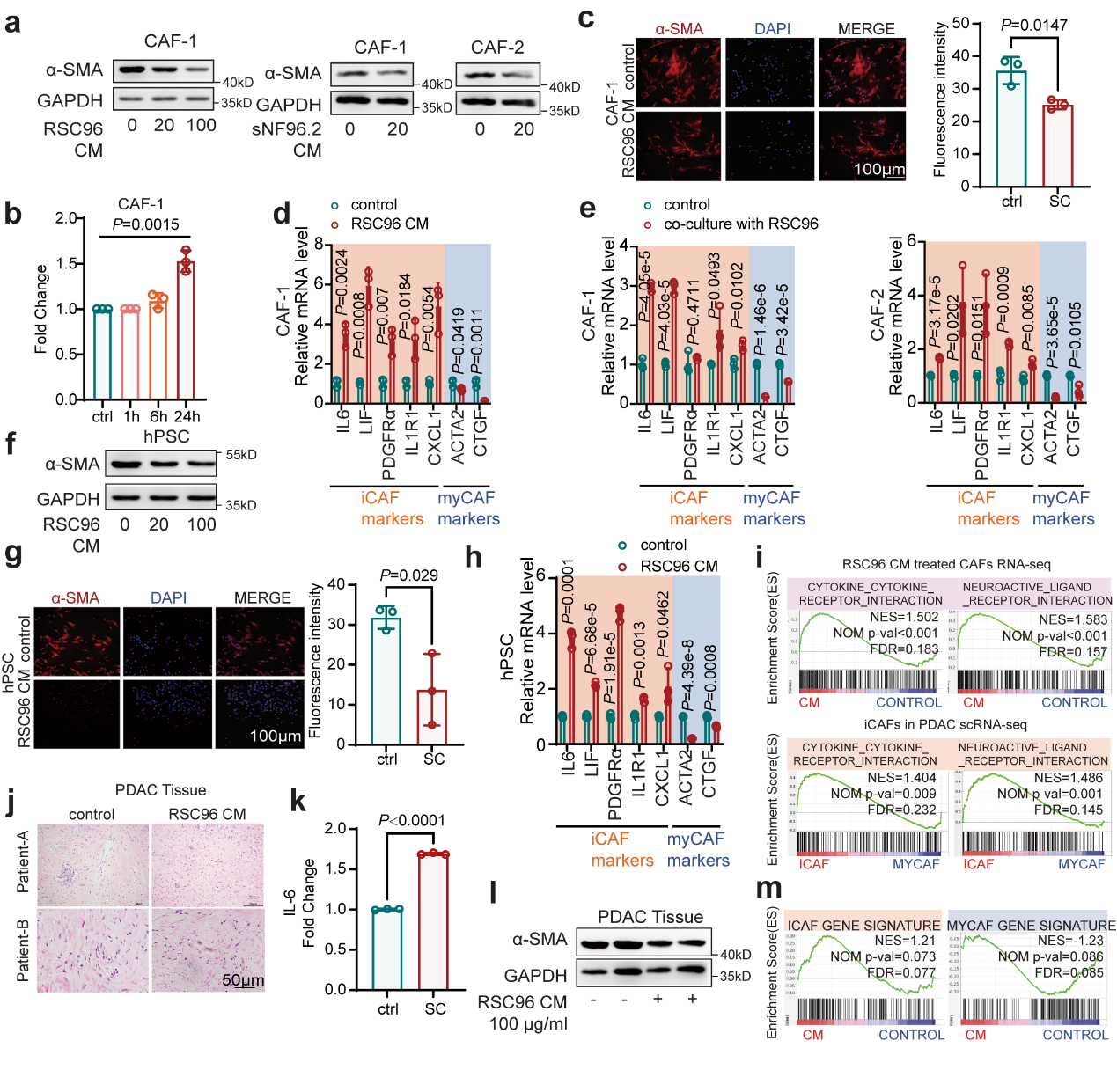


**Supplementary Figure S7. Schwann Cells Induce Phenotypic Switch in CAFs**

**a.** The protein levels of α-SMA in CAFs cultured with 0, 20 or 100 μg/ml RSC96 or 0, 20 μg/ml sNF96.2 CM.

**b.** The fold change of IL-6 in CAF-1 cultured with RSC96 CM for 0, 1, 6, or 24 hours was evaluated by ELISA. The concentration of IL-6 at 0 h was used as internal control.

**c.** Representative IF image with anti-α-SMA in CAF-1 cultured with RSC96 CM and control. Scale bar, 100µm.

**d, e.** Relative mRNA levels of iCAF and myCAF markers in CAFs cultured with RSC96 CM (**d**) or co-culture with RSC96 cells (**e**) compared to control group.

**f.** The protein level of α-SMA in hPSCs cultured with 0, 20 or 100 μg/ml RSC96 CM.

**g.** Representative image of IF with anti-α-SMA in hPSCs cultured with RSC96 CM or control. Scale bar, 100µm. c, g, Images are representative of 3 biologically independent experiments with CAF-1/hPSC cells with similar results.

**h.** Relative mRNA levels of iCAF and myCAF markers in hPSCs cultured with RSC96 CM compared to control group.

**i.** GSEA plot showing cytokine-cytokine receptor interaction and neuroactive ligand receptor interaction pathway was enriched in RSC96 CM induced CAFs and putative iCAFs cluster identified by scRNA-seq.

**j.** H&E staining showing the histological features of PDAC tissues in an *ex vivo* culture model. Images are representative of 3 biologically independent PDAC samples with similar results. Scale bar, 50µm.

**k.** The fold change of IL-6 in PDAC tissues after RSC96 CM incubation was evaluated by ELISA.

**l.** Protein levels of α-SMA in PDAC tissues incubated with RSC96 CM. a, f, l, Data are representative of n = 2 independent experiments.

**m.** GSEA plot showing the enrichment score (ES) of iCAF and myCAF signatures in CAFs. Pathway enrichment analysis was performed based on bulk RNA-seq of RSC96 CM induced CAFs and controls. i, m, NES normalized enrichment score, corrected for multiple comparisons using FDR method, *P*-value were showed in plots.

b-e, g, h, k, Data are the mean ± s.d. of n = 3 independent experiments.

Statistical analysis: unpaired two-sided *t*-test (b-e, g, h, k). Source data are provided as a Source Data file.

**
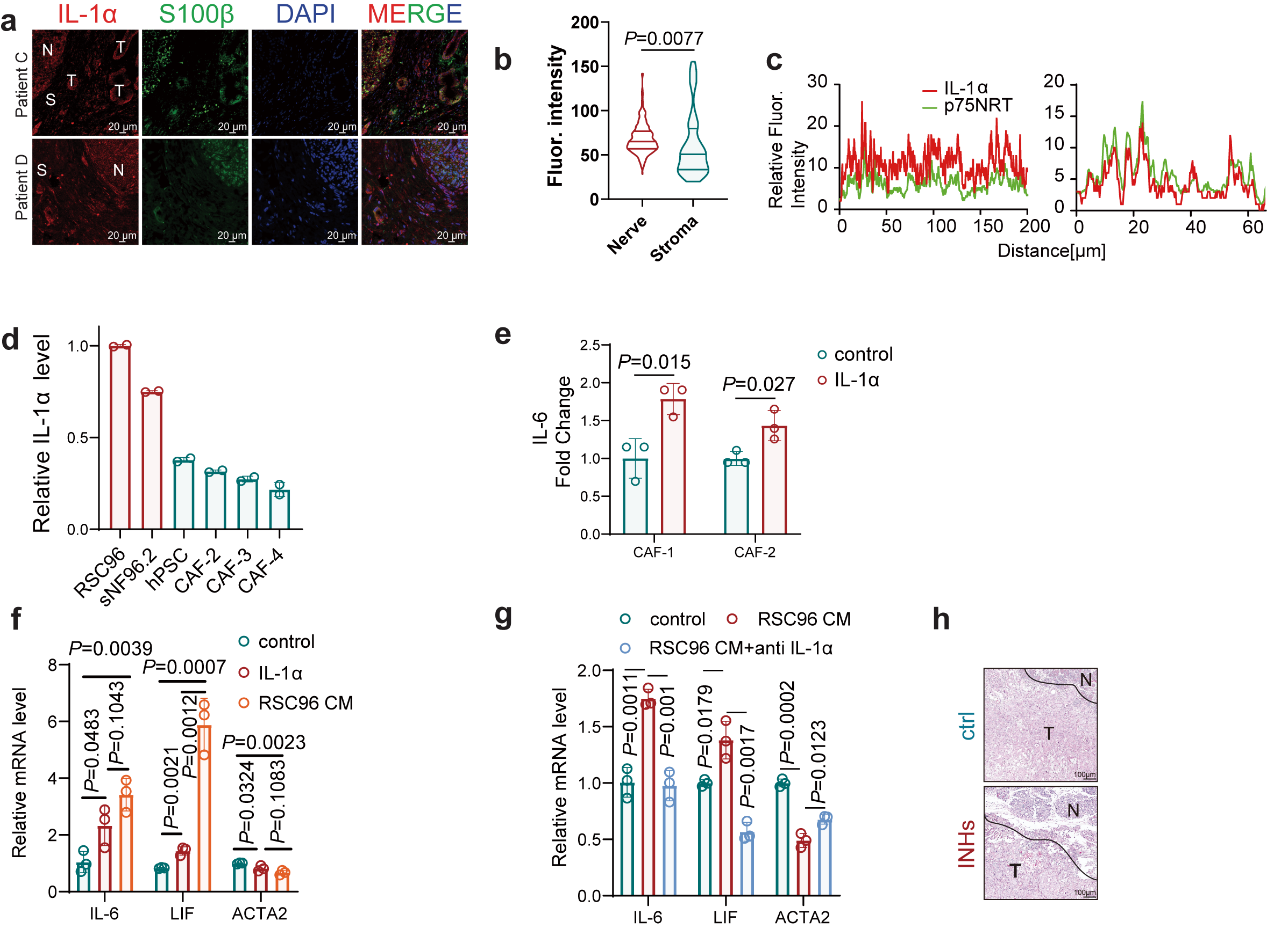
**

**Supplementary Figure S8. Schwann Cells Induce Phenotypic Switch in CAFs via IL-1α**

**a.** Representative images of IF with anti-IL-1α and S100β in the neuro-stroma niche. Scale bar, 20µm. Images are representative of 5 biologically independent PDAC samples with similar results. N, nerves; T, tumor; S, stroma.

**b.** Quantification of IL-1α intensity in the nerve or stroma regions in patient D. For each violin, the minimum, first quartile, median, third quartile, and maximum were displayed.

**c.** Profile intensities of IL-1α and p75NRT on the white arrows in PDAC patients in Figure 6j.

**d.** The relative levels of IL-1α in hPSC, CAFs, RSC96 and sNF96.2 CM was evaluated by ELISA.

**e.** Fold change of IL-6 in CAFs treated with IL-1α was evaluated by ELISA. No treatment was used as an internal control.

**f.** Relative mRNA levels of iCAF and myCAF markers in CAF-1 treated with IL-1α or RSC96-CM.

**g.** The relative mRNA levels of iCAF and myCAF markers in CAF-1 cultured by RSC96 CM pre-treated with neutralizing antibodies against IL-1α or IgG control.

**h.** Representative H&E staining of orthotopic tumors induced by co-injection of CAFs, CFPAC-1 and sNF96.2. N, normal pancreas; T, tumor. Images are representative of 5 biologically independent animals with similar results. Scale bar, 100µm.

e-g, Data are the mean ± s.d. of n = 3 independent experiments; d, Data are the mean ± s.d. of n = 2 independent experiments.

Statistical analysis: unpaired two-sided *t*-test (b, e-g). Source data are provided as a Source Data file.


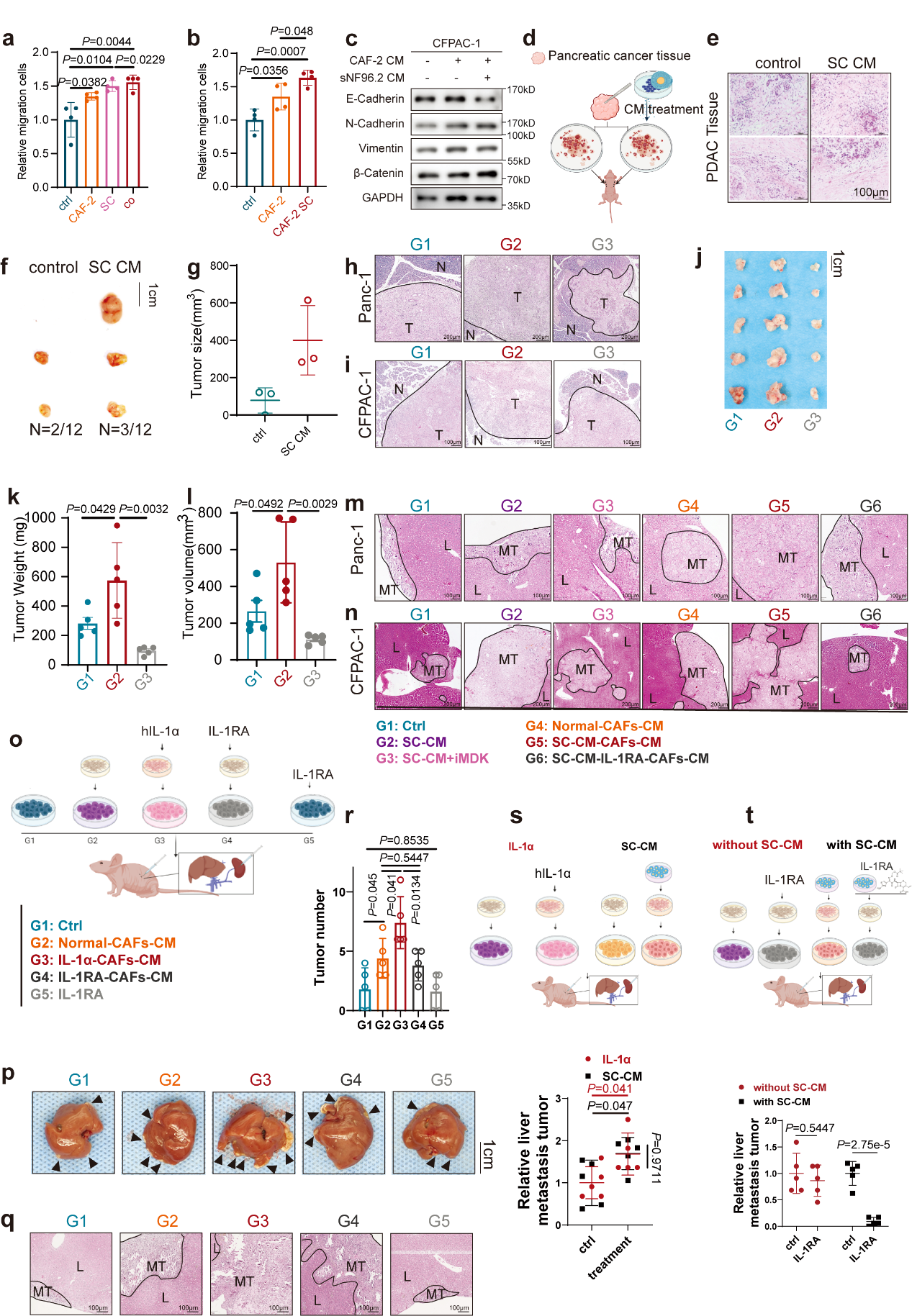


**Supplementary Figure S9. Schwann Cells Hijack iCAFs to Accelerate Cancer Progression**

**a, b.** The migration capabilities of CFPAC-1 cells were enhanced by the CM of CAFs, which were directly co-cultured (**a**) or cultured with Schwann cells CM (**b**).

**c.** Protein levels in CFPAC-1 cells treated with CM of CAF-2 cultured with or without RSC96 CM. Data are representative of n = 3 independent experiments.

**d.** Schematic diagram of the *in vivo* PDX model. PDAC tissues were incubated with RSC96 CM *in vitro* and subcutaneously implanted into the dorsal flanks of mice (n=12).

**e.** H&E staining showing the histological features of PDAC tissue used for the PDX model. Scale bar, 100µm. Images are representative of 3 biologically independent PDAC samples with similar results.

**f, g.** Photograph (**f**) of subcutaneous PDX (n=2/12 vs n=3/12) and the final tumor volumes (**g**) summarized in a dot chart.

**h, i,** Representative H&E staining of orthotopic tumors induced by co-injection of CAFs with Panc-1(**h**) or CFPAC-1(**i**). Scale bar, 200µm (h) and 100µm (i).

**j-l.** Photograph (**j**) and quantification (**k, l**) of orthotopic injection model with co-injection of CAFs and CFPAC-1.

**m, n.** Representative H&E staining of *in vivo* liver metastatic model with Panc-1(**m**) or CFPAC-1(**n**). L, liver; MT, metastasis tumor. Scale bar, 100µm (m) and 200µm (n).

**o.** Schematic diagram of controls for *in vivo* liver metastatic model. CFPAC-1 was pre-treated with ctrl medium (G1), ctrl-CAF-CM(G2), IL-1α-induced CAF-CM(G3), IL-1RA-induced CAF-CM (G4) or IL-1α only (G5), then injected via spleen (n=5).

**p-r.** Representative photograph (**p**), H&E staining (**q**) and quantification (**r**) of CFPAC-1 liver metastatic tumors. Scale bar, 100µm. h, i, m, n, q, Images are representative of 5 biologically independent animals with similar results.

**s.** Dot plot shows the relative liver metastatic tumors formed by CFPAC-1 cultured with CM of IL-1α or SC-CM activated CAF.

**t.** Dot plot shows the relative liver metastatic tumors formed by CFPAC-1 treated with

CM of IL-1RA or SC-CM-IL-1RA treated CAF. s, t, The treatment groups were normalized to their controls in every condition.

a-b, Data are the mean ± s.d. of n = 4 independent experiments; g, Data are the mean ± s.d. of n = 3 independent experiments; k, l, r-t, Data are the mean ± s.d. of n = 5 independent experiments. d, o, s, t, Created with BioRender.com.

Statistical analysis: unpaired two-sided *t*-test (a, b, k, l, r-t). Source data are provided as a Source Data file.


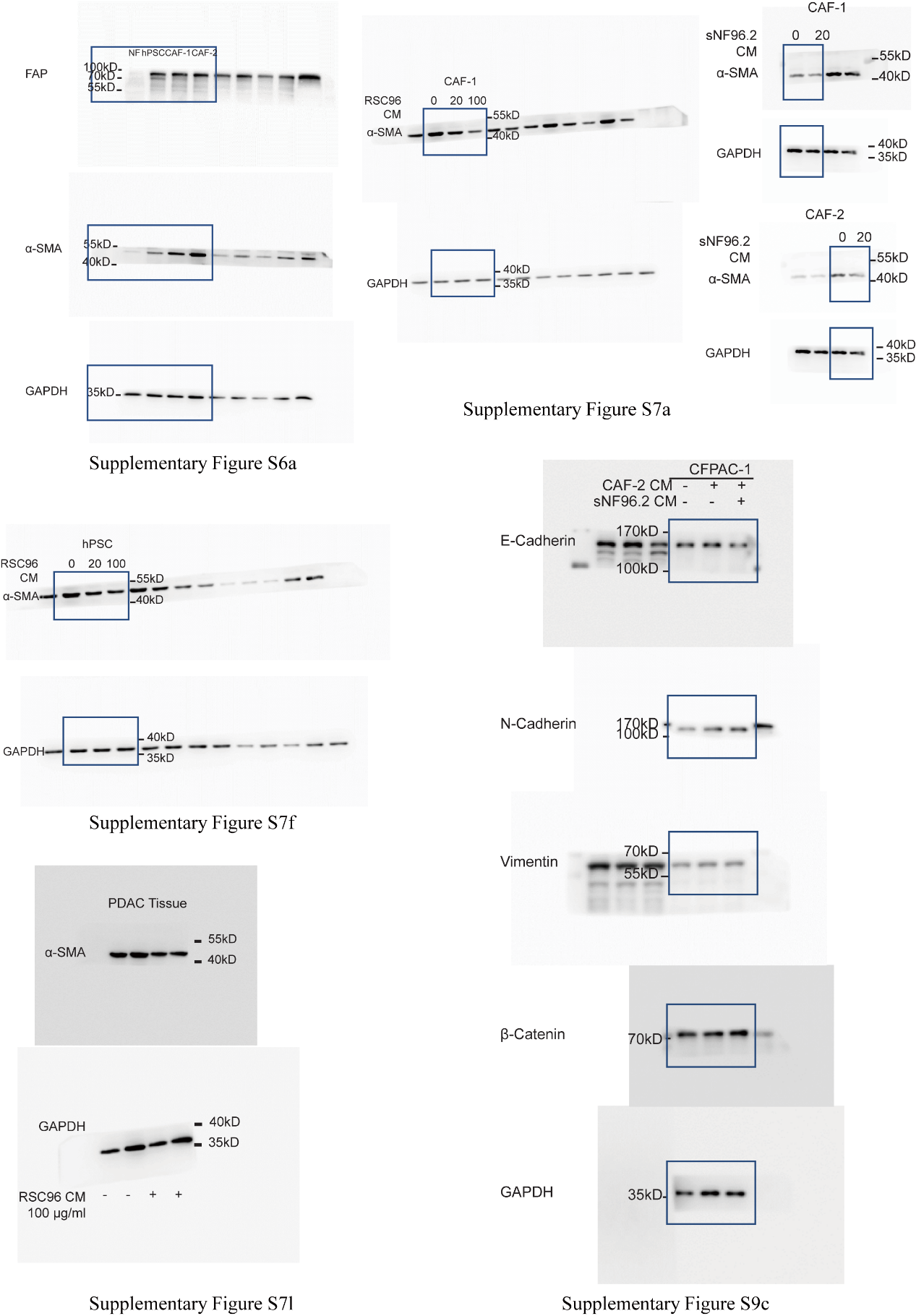


**Supplementary Figure S10.** Uncropped blot images in Supplementary Figures

**Supplementary Table S1.** Marker genes in bulk RNA-seq used to validated in ST data

| cancer cell bulk RNA-seq signature | |  | CAFs bulk RNA-seq signature | |
| --- | --- | --- | --- | --- |
| Control | SC-CM associated |  | Control | SC-CM associated |
| MUC13 | TIMP2 |  | PHACTR3 | FOSB |
| MMP3 | SCEL |  | DAW1 | FOS |
| GDF15 | COL1A1 |  | FAM83E | NGFR |
| CYP4F3 | MT2A |  | GABRP | GXYLT2 |
| CTDSPL | ANO1 |  | CCL28 | DUSP15 |
| SPINK1 | HAS2 |  | FOXA3 | LZTS1 |
| CLRN3 | MDM4 |  | B3GALT2 | MSC |
|  | DCTN5 |  | FGFBP1 | SRGAP3 |
|  |  |  | RXFP1 | CES3 |
|  |  |  | PRSS8 | FLG |
|  |  |  | CDH16 | SLC16A9 |
|  |  |  | PLAAT2 | SH3TC1 |
|  |  |  | PCDHB5 | SRPX |
|  |  |  | AP1M2 | NPAS3 |
|  |  |  | GMNC | NDUFA6 |
|  |  |  | IL19 | OLFML2B |
|  |  |  | MARCO | PTK6 |
|  |  |  | ACTBL2 | RXFP4 |
|  |  |  | STEAP4 | MMP19 |
|  |  |  | GRHL2 | GDF15 |

**Supplementary Table S2.** The primer sequences used in this study

| **Primer** | **Sequence(5’-3’）** |
| --- | --- |
| IL1R1 F | GGCTGAAAAGCATAGAGGGAAC |
| IL1R1 R | CTGGGCTCACAATCACAGG |
| CTGF F | CAGCATGGACGTTCGTCTG |
| CTGF R | AACCACGGTTTGGTCCTTGG |
| IL6 F | ACTCACCTCTTCAGAACGAATTG |
| IL6 R | CCATCTTTGGAAGGTTCAGGTTG |
| LIF F | CCAACGTGACGGACTTCCC |
| LIF R | TACACGACTATGCGGTACAGC |
| PDGFRA F | TTGAAGGCAGGCACATTTACA |
| PDGFRA R | GCGACAAGGTATAATGGCAGAAT |
| ACTA2 F | CAGGGCTGTTTTCCCATCCAT |
| ACTA2 R | GCCATGTTCTATCGGGTACTTC |
| GAPDH F | ACAACTTTGGTATCGTGGAAGG |
| GAPDH R | GCCATCACGCCACAGTTTC |
| Vimentin F | TTGCCGTTGAAGCTGCTAACTACC |
| Vimentin R | AATCCTGCTCTCCTCGCCTTCC |
| E-cadherin F | AGTCACTGACACCAACGATAAT |
| E-cadherin R | ATCGTTGTTCACTGGATTTGTG |
| N-cadherin F | CGATAAGGATCAACCCCATACA |
| N-cadherin R | TTCAAAGTCGATTGGTTTGACC |
| CXCL1 F | AACCGAAGTCATAGCCACAC |
| CXCL1 R | GTTGGATTTGTCACTGTTCAGC |
