# Supplementary material for: Schwann cells regulate tumor cells and cancer-associated fibroblasts in the pancreatic ductal adenocarcinoma microenvironment
Source: Nat Commun. 2023 Jul 31;14:4600. doi: 10.1038/s41467-023-40314-w (PMC10390497; doi:10.1038/s41467-023-40314-w)
Supplement: Supplementary file 2 — Description of Additional Supplementary Files [file 41467_2023_40314_MOESM2_ESM.pdf]

Description of Additional Supplementary Files

File Name: Supplementary Data 1

Description: Marker genes in scRNA-seq

File Name: Supplementary Data 2

Description: Marker genes in spatial transcriptomics data

File Name: Supplementary Data 3

Description: Proteins in Schwann cell CM via mass spectrometry (MS)
